# Prescription Patterns for Pulmonary Vasodilators in the Treatment of Pulmonary Hypertension Associated With Chronic Lung Diseases: Insights From a Clinician Survey

**DOI:** 10.3389/fmed.2021.764815

**Published:** 2021-12-03

**Authors:** Christopher A. Thomas, Justin Lee, Roberto J. Bernardo, Ryan J. Anderson, Vladimir Glinskii, Yon K. Sung, Kristina Kudelko, Haley Hedlin, Andrew Sweatt, Steven M. Kawut, Rishi Raj, Roham T. Zamanian, Vinicio de Jesus Perez

**Affiliations:** ^1^Department of Pulmonary, Allergy and Critical Care Medicine, Stanford University, Stanford, CA, United States; ^2^Quantitative Sciences Unit, Department of Medicine, Stanford University, Stanford, CA, United States; ^3^Division of Pulmonary, Critical Care and Sleep Medicine, The University of Oklahoma Health Sciences Center, Tulsa, OK, United States; ^4^Pulmonary, Allergy and Critical Care Medicine Division, Perelman School of Medicine at the University of Pennsylvania, Philadelphia, PA, United States

**Keywords:** pulmonary hypertension, chronic lung disease, vasodilators, survey, group 3 PH

## Abstract

**Background:** Pulmonary hypertension is a complication of chronic lung diseases (PH-CLD) associated with significant morbidity and mortality. Management guidelines for PH-CLD emphasize the treatment of the underlying lung disease, but the role of PH-targeted therapy remains controversial. We hypothesized that treatment approaches for PH-CLD would be variable across physicians depending on the type of CLD and the severity of PH.

**Methods and Results:** Between May and July 2020, we conducted an online survey of PH experts asking for their preferred treatment approach in seven hypothetical cases of PH-CLD of varying severity. We assessed agreement amongst clinicians for initial therapy choice using Fleiss' kappa calculations. Over 90% of respondents agreed that they would treat cases of severe PH in the context of mild lung disease with some form of PH-targeted therapy. For cases of severe PH in the context of severe lung disease, over 70% of respondents agreed to use PH-targeted therapy. For mild PH and mild lung disease cases, <50% of respondents chose to start PH-specific therapy. There was overall poor agreement between respondents in the choice to use mono-, double or triple combination therapy with PH-specific agents in all cases.

**Conclusion:** Although management guidelines discourage the routine use of PH-targeted therapies to treat PH-CLD patients, most physicians choose to treat patients with some form of PH-targeted therapy. The choice of therapy and treatment approach are variable and appear to be influenced by the severity of the PH and the underlying lung disease.

## Introduction

Pulmonary hypertension (PH) associated with chronic lung disease (PH-CLD) is a subgroup of group 3 PH associated with significant morbidity and mortality (1, 2). Although PH-CLD is relatively common, the pathophysiology is quite diverse and complex, and even mild PH in the setting of CLD has been associated with reduced functional status and worse outcomes (3). Diagnosis of PH-CLD remains challenging even for the most experienced medical professionals. When patients have a diagnosis of CLD and PH, the clinician must use data from lung imaging, pulmonary function tests, and right heart catheterization (RHC) to confirm the diagnosis and phenotype of patients according to disease severity. As such, PH-CLD presents many diagnostic and therapeutic challenges to even the most experienced clinicians.

Current consensus guidelines recommend treating the primary lung disease and advise against routine use of PH-targeted drugs in PH-CLD (1). However, clinicians may favor treating PH-CLD empirically with pulmonary vasodilators, although the clinical benefit of monotherapy or combination therapy in this setting remains controversial. In April 2021, following the results of the INCREASE study, inhaled treprostinil became the first and only drug approved by the Food and Drug Administration (FDA) for the treatment of PH associated with interstitial lung disease (ILD) (4). For other PH-targeted therapies, the data remains controversial and may even indicate that certain drugs increase the risk of complications in PH-CLD. For example, the use of ambrisentan and riociguat is contraindicated in patients with PH associated with idiopathic pulmonary fibrosis (IPF) and idiopathic interstitial pneumonia based on clinical studies demonstrating an increased incidence of morbidity related to these drugs (5, 6). As such, it is advisable to avoid empirical use of endothelin receptor antagonists and riociguat in PH-CLD.

At present, there is limited data on the practice patterns of physicians who treat PH-CLD in terms of their approach to using PH-targeted agents. A 2015 survey of treatment practices for non-Group 1 PH patients at national PH referral centers reported that 80% of Group 3 PH received PH-targeted therapy (7). Since then, there has been no reassessment to determine whether the attitudes toward PH-CLD have changed following the publication of the 2018 6^th^ World Symposium on PH (6^th^ WSPH) proceedings (8). Moreover, despite the safety concerns with use of endothelin receptor antagonists or riociguat (5, 6), it is unclear whether physicians rely on these drugs when choosing to empirically treat patients with CLD or whether they favor the use of mono- or combination therapy in a manner similar to the current standard of care for Group 1 PH patients.

Given the lack of consensus regarding the use of PH-targeted therapies in CLD, we hypothesized that agreement to treat with PH-targeted drugs would be higher in cases of severe PH-CLD and that use of monotherapy would be preferred over combination therapy. Our goal was to understand the current attitudes among PH professionals regarding the treatment of PH-CLD and how disease severity dictates the decision to choose a specific approach to treatment.

It must be noted that this study reflects the attitude of physicians toward the use of PH medications to treat PH-CLD prior to the publication of the INCREASE trial and approval of inhaled treprostinil for PH-ILD in April 2021.

## Methods

We created an online survey to gather information on the treatment preferences of clinicians who manage PH-CLD. The survey was created using the Qualtrics survey tool provided by Stanford University. The survey was reviewed by the Stanford University Institutional Review Board and granted exempt status. The survey included eight demographic questions on clinician training backgrounds, experience with PH-CLD, and institution location and type (see Appendix I for survey details). We submitted the survey for dissemination to the following organizations' email listservs: the Pulmonary Hypertension Clinicians and Researchers (PHCR) network, the American College of Chest Physicians (ACCP), and the American Thoracic Society (ATS) pulmonary circulation assembly. We chose these listservs because they are well-known organizations with broad distribution to the PH clinician community. The survey was conducted between May and July 2020, and respondents were asked to complete the survey only once. Responses were anonymous, and all data was stored in the secure online Qualtrics database.

**Figure 1:**
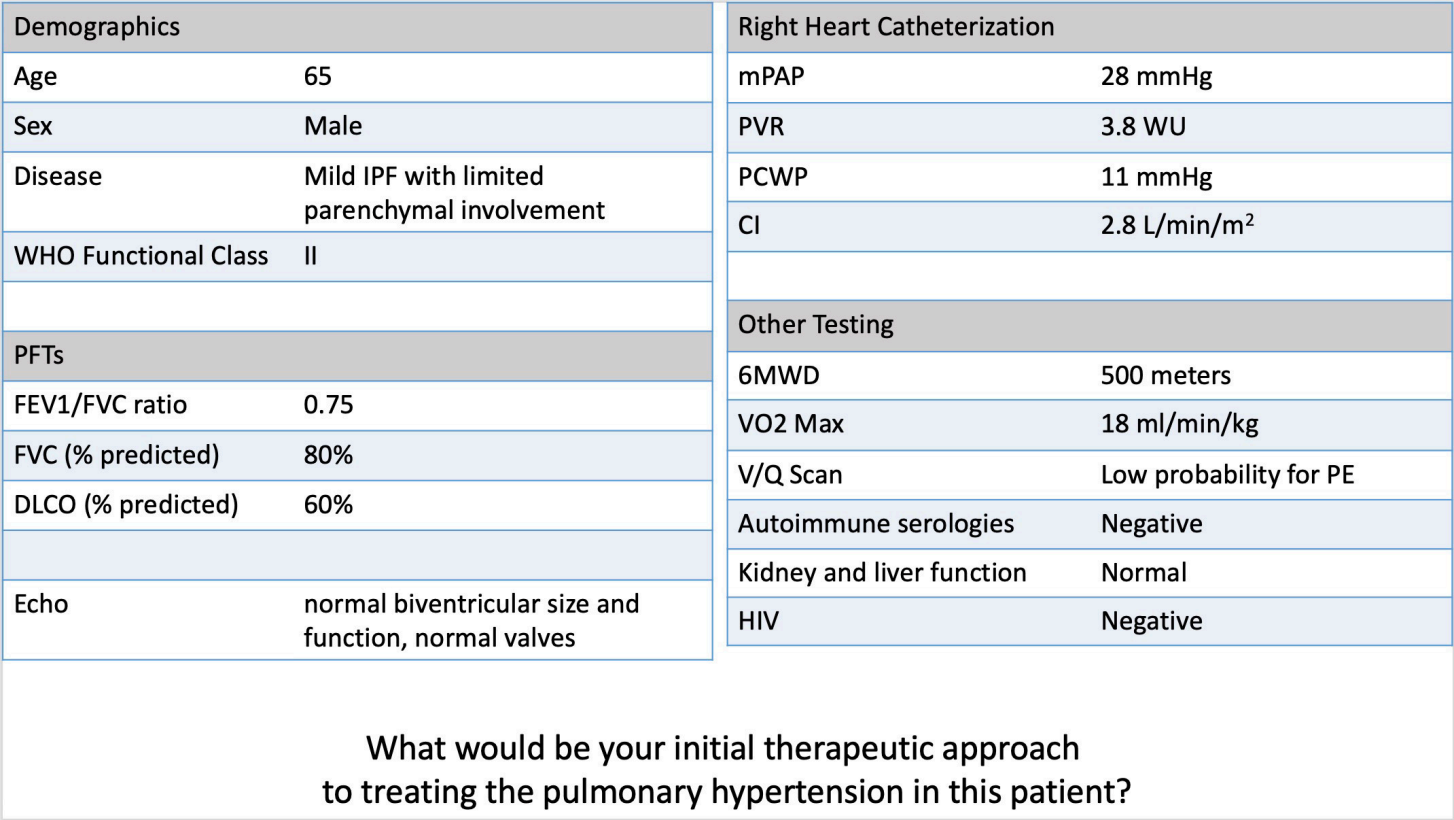
Example PH-CLD case from the survey (case 1—Mild IPF/Mild PH).

**Figure 2:**
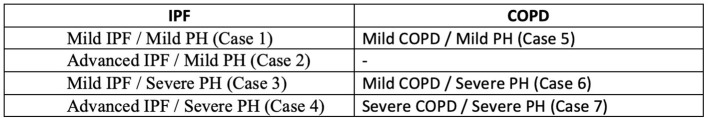
PH-CLD phenotypes for each of the seven hypothetical cases. Severe PH was defined as a mPAP > 35 mmHg, severe IPF was defined as FVC < 70%, and severe COPD was defined as FEV1 <60%. These values were based on the 6th WSPH Group 3 task force recommendations (1).

The survey included seven hypothetical cases designed by PH and ILD experts at Stanford University. We chose to focus the clinical cases on IPF and chronic obstructive pulmonary disease (COPD) as these two disease entities are commonly representative of PH-CLD. Each case included the patient's age, biological sex, CLD diagnosis, WHO functional class, pulmonary function test (PFT) values, echocardiography results, RHC hemodynamics, and 6-min walk distance (6MWD) (see Figure 1 for an example case, and the Appendix for the survey including all cases). Cases were based on the current definitions of PH-CLD from the 6th World Symposium on Pulmonary Hypertension (1): (1) *CLD without PH*: mPAP <21 mmHg, or mPAP 21–24 with PVR <3 WU, (2) *CLD with mild PH*: mPAP 21–24 mmHg with PVR > 3 WU, or mPAP 25–34, (3) *CLD with severe PH*: mPAP > 35 mmHg, or mPAP > 25 mmHg and low cardiac index (CI <2.0 l/min/m^2^). The cases were created using different combinations of mild to severe CLD and PH and designed to be straightforward regarding the severity of both the lung disease and the PH. Four cases were created with “severe” PH, defined as an mPAP > 35 mmHg. Of these cases, two were paired with a “mild” CLD diagnosis (i.e., Group 1 PH-like phenotype), and another two were “severe” PH paired with advanced CLD. The case designs are summarized in Figure 2.

We included other variables to make the overall clinical picture clear and to eliminate the possibility of other etiologies of PH [i.e., connective tissue diseases (CTD) or chronic thromboembolic pulmonary hypertension (CTEPH)]. To keep the survey as short as possible, we chose not to include the combination of severe COPD and mild PH because the treatment choice variability was likely to be relatively low (i.e., most would choose no PH-targeted treatment).

After reading the cases, clinicians were first asked, “What would be your initial therapeutic approach to treating pulmonary hypertension in this patient?” The answer choices for this question were “no medical therapy,” “single-agent therapy,” “double agent therapy,” or “triple agent therapy.” If they chose no medical therapy, the survey proceeded to the next case. If they decided on single, double or triple agent therapy, respondents were asked to pick a specific combination therapy regimen [e.g., phosphodiesterase-5 inhibitor (PDE5 inhibitor), endothelin receptor antagonist (ERA), or prostacyclin analog]. See the Appendix for complete answer choices.

The primary analysis was agreement amongst clinicians for initial therapy choice (i.e., 0 vs. 1 vs. 2 vs. 3 drug therapy) for each of the 7 cases. This analysis was done using Fleiss' kappa calculations (9) amongst all survey respondents who answered all seven cases. As a sensitivity analysis, Fleiss' kappa was also calculated amongst US practicing clinicians. Additionally, Fleiss' kappa was calculated for the subset of IPF and COPD cases. Finally, Fleiss' kappa was calculated separately for both pulmonologists and cardiologists. Fleiss' kappa values can range from −1 to 1, with negative values indicating more disagreement than expected by chance, positive values below 0.40 suggesting poor agreement beyond what is expected by chance, and values >0.75 suggesting excellent agreement (10). All analyses were conducted with R version 3.5.1, and the “likert” R package (11) version 1.3.5 was used to plot responses by case.

## Results

**Figure 3:**
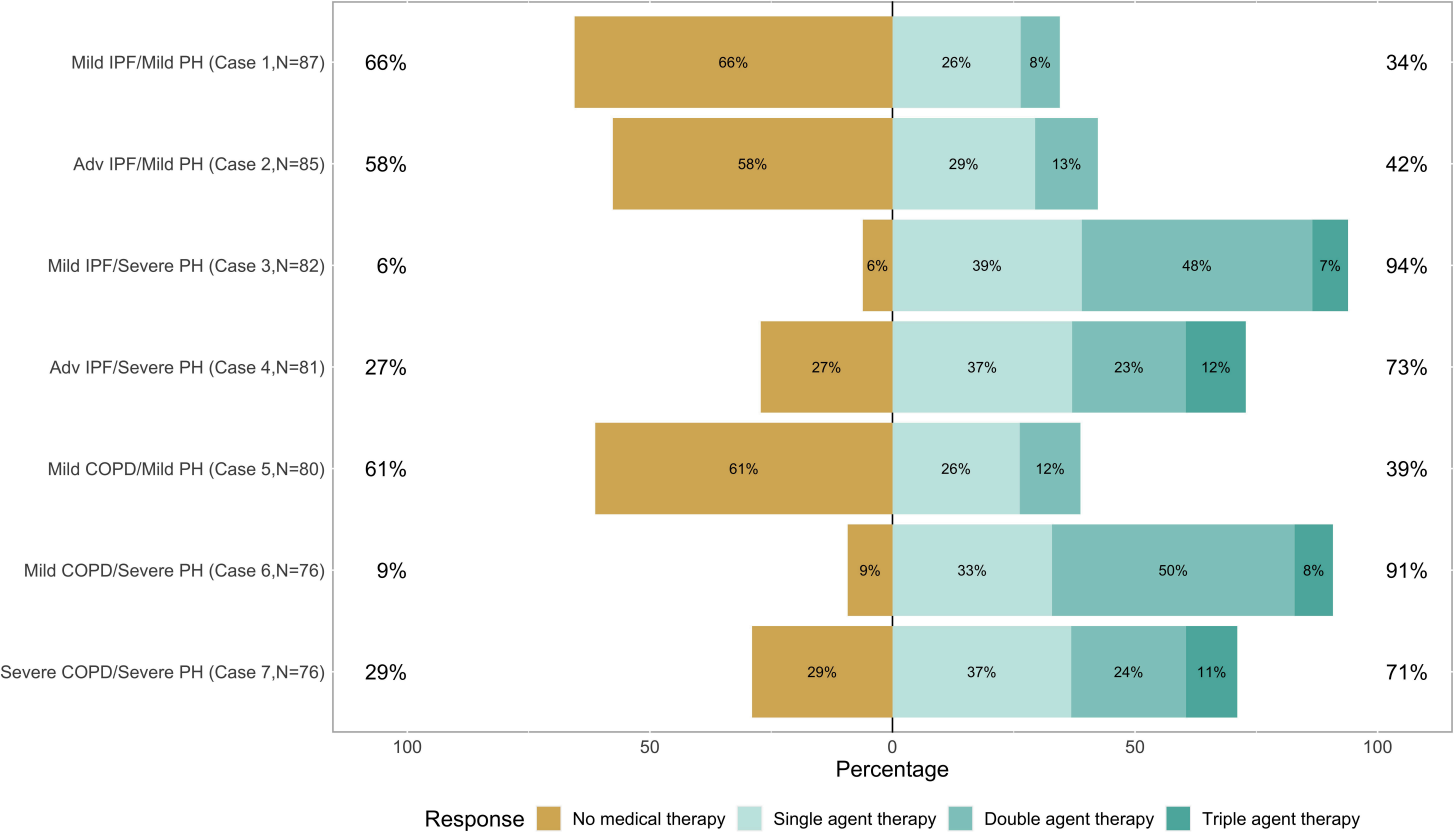
Plot of the responses to each of the seven cases.

**Figure 4:**
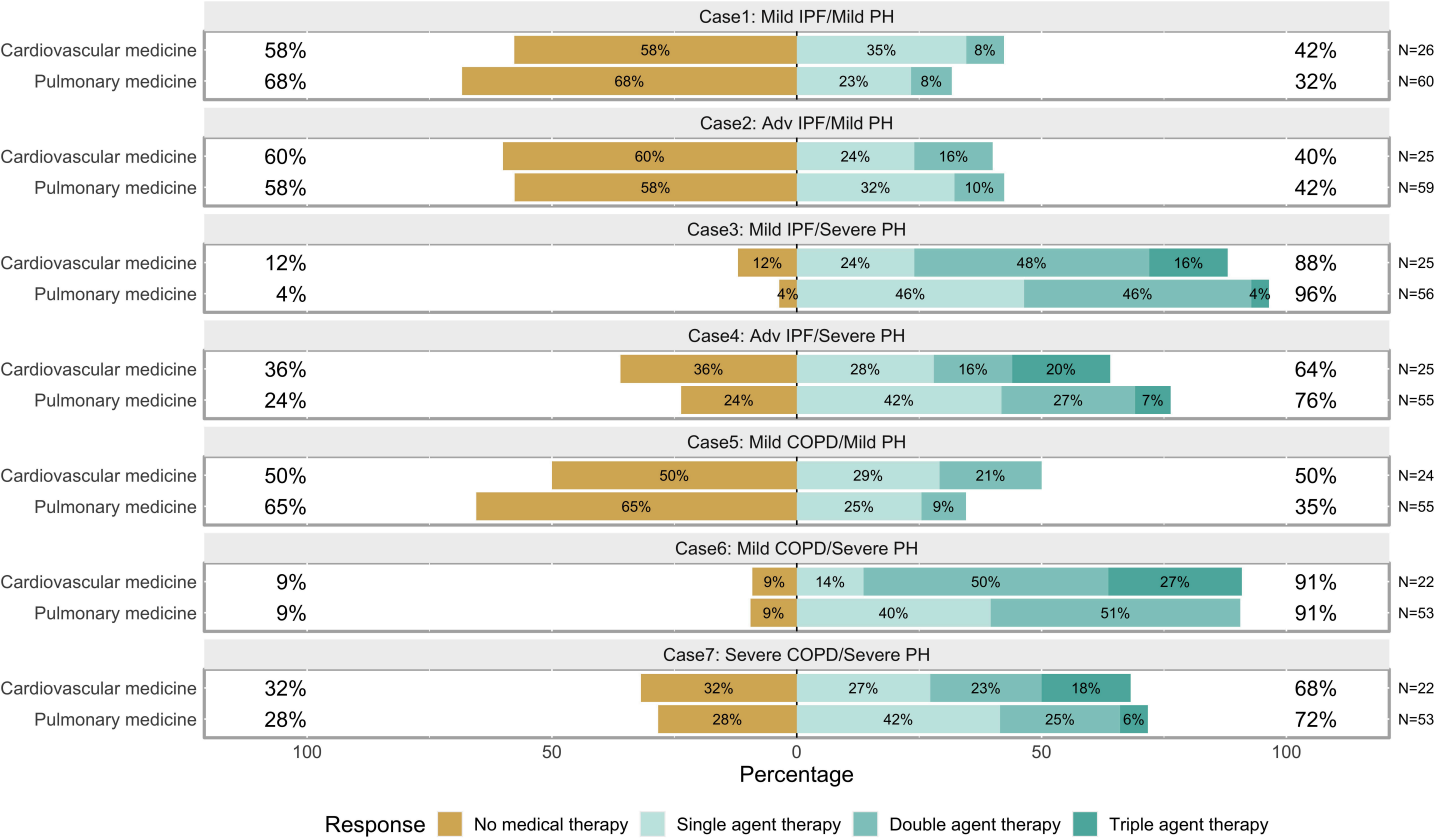
Plot of the responses to each of the seven cases by specialty.

Demographic characteristics for respondents who responded to at least one of the cases are shown in Table 1. Hundred clinicians responded to at least one survey question, 87 clinicians responded to at least 1 case, and 76 clinicians completed the entire survey. Though we disseminated the survey to three international organizations, most respondents (92%) reported practicing in the United States. Responses provided for each of the seven cases were analyzed globally (Figure 3) and specialty (Figure 4).

**Table 1:** Demographic characteristics of the survey respondents.

**Demographics of those who answered at least one case (*N* = 87)**	
Title (%)	
Attending physician/consultant	84 (96.6%)
Physician-in-training	1 (1.1%)
Nurse practitioner	2 (2.3%)
Training background (%)	
Pulmonary medicine training	60 (69%)
Cardiology medicine training	26 (29.9%)
Other clinicians	1 (1.1%)
Years in practice [median (IQR)]	12 [6–20]
Practicing in the United States (%)	80 (92%)
Institution type (%)	
Pulmonary hypertension center of comprehensive care	39 (48.8%)
Pulmonary hypertension regional clinical program	4 (5.0%)
Academic center without PHA designation	28 (35.0%)
Community practice without PHA designation	7 (8.8%)
Other	2 (2.5%)
Percentage of time spent practicing clinical medicine [%, median (IQR)]	75.0 [60.0–81.0]
Percentage of time spent treating pulmonary hypertension patients [%, median (IQR)]	50.0 [30.0–76.0]
Unique group 3 pulmonary hypertension patients in the past year of practice [#, median (IQR)]	30.0 [20.0–50.0]

*IQR, interquartile range*.

In cases with severe PH (regardless of the severity of the lung disease), clinicians were more likely to choose some form of PH therapy (71–94%) rather than no therapy. Over half (58–66%) of the clinicians chose no PH therapy in the three cases with mild PH. In the “Group 1-like" cases [Case 3 (mild IPF and severe PH) and Case 6 (mild COPD and severe PH)], more than 90% of clinicians chose some form of PH treatment. In Case 3, 39% of clinicians chose single-agent therapy, 48% chose double agent therapy, and 7% chose triple agent therapy, for a total of 94% of clinicians selecting some form of PH therapy. In Case 6, 33% of clinicians chose single-agent treatment, 50% chose double agent therapy, and 8% chose triple agent therapy, for a total of 91% of clinicians selecting some form of PH therapy.

In the other cases [Case 4 (advanced IPF and severe PH) and Case 7 (severe COPD and severe PH)], more than 70% of clinicians chose some form of therapy. In Case 4, 37% of clinicians chose single-agent treatment, 23% chose double agent therapy, and 12% chose triple treatment, for a total of 73% of clinicians chose some form of PH therapy. In Case 7, 37% of clinicians chose single-agent treatment, 24% chose double agent therapy, and 11% chose triple treatment, for a total of 71% of clinicians selecting some sort of PH therapy.

In the cases of mild PH [Case 1 (mild IPF and mild PH), Case 2 (advanced IPF and mild PH), and Case 5 (mild COPD and mild PH)], over half (58–66%) of the clinicians chose not to start PH therapy.

### Fleiss' Kappa Analyses

Fleiss' kappa calculation for the overall analysis yielded a value of 0.12 (Table 2). This value is consistent with poor overall agreement among the 76 clinicians who completed all 7 cases regarding choosing between the number of therapies (i.e., 0, 1, 2, or 3 PH therapies). Agreement for IPF specific case questions (Cases 1, 3, and 4) and COPD-specific case questions (Cases 5, 6, and 7) was also poor with kappa values of 0.13 and 0.10, respectively (Table 2). We excluded Case 2 (advanced IPF and mild PH) from this analysis because its corresponding case (severe COPD and mild PH) was not included in the survey. The Fleiss' kappa for survey respondents who reported practicing in the US and who completed the entire survey (*n* = 70) was similarly low (0.11) for all cases, which is consistent with poor agreement among US clinicians (Table 3). We also calculated Fleiss' kappa for pulmonologists (*n* = 53) and cardiologists (*n* = 23) who completed the entire survey (Tables 4, 5). The Fleiss' kappa for pulmonologists (0.13) was slightly higher than that of all survey respondents, and the Fleiss' kappa for the cardiologists (0.07) was slightly lower than that for all survey respondents.

**Table 2:** Fleiss' Kappa calculations for all respondents who completed the entire survey (*n* = 76).

**Measuring agreement on:**	**Number of raters**	**Kappa: all cases**	**Kappa: IPF cases only**	**Kappa: COPD cases only**
Number of therapies (0, 1, 2, 3)	76	0.12	0.13	0.10
None vs. any therapy	76	0.23	0.27	0.21

**Table 3:** Fleiss' Kappa calculations for US respondents who completed the entire survey (*n* = 70).

**Measuring agreement on:**	**Number of raters**	**Kappa: all cases**	**Kappa: IPF cases only**	**Kappa: COPD cases only**
Number of therapies (0, 1, 2, 3)	70	0.11	0.12	0.10
None vs. any therapy	70	0.24	0.28	0.23

**Table 4:** Fleiss' Kappa calculations for respondents who identified as pulmonologists and completed the entire survey (*n* = 53).

**Measuring agreement on:**	**Number of raters**	**Kappa: all cases**	**Kappa: IPF cases only**	**Kappa: COPD cases only**
Number of therapies (0, 1, 2, 3)	53	0.13	0.14	0.12
None vs. any therapy	53	0.25	0.31	0.23

**Table 5:** Fleiss' kappa for survey respondents who identified as cardiologists and completed the entire survey (*n* = 22).

**Measuring agreement on:**	**Number of raters**	**Kappa: all cases**	**Kappa: IPF cases only**	**Kappa: COPD cases only**
Number of therapies (0, 1, 2, 3)	22	0.07	0.07	0.05
None vs. any therapy	22	0.14	0.14	0.12

### Individual Medication Choices for Severe PH Cases

**Figure 5:**
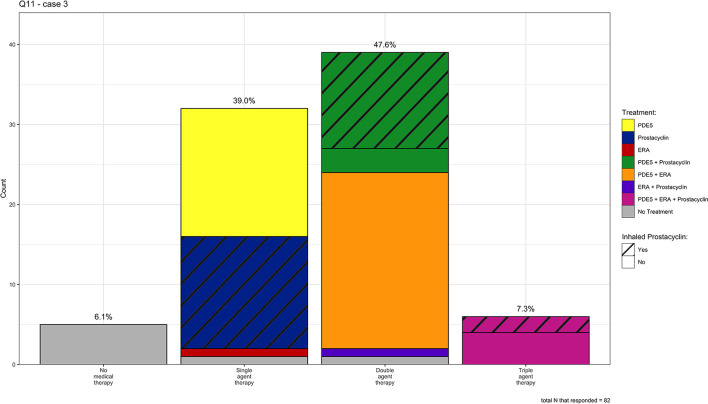
Individual medication choices for case 3 (mild IPF and severe PH), *N* = 82.

We describe Cases 3 and 6 (mild lung disease with severe PH) as a “Group 1-like” phenotype. In Case 3 (mild IPF and severe PH), 39, 48, and 7% of clinicians chose single, double, and triple agent therapy, respectively. Of those clinicians who chose single-agent therapy (*n* = 32), 52% chose a PDE5 inhibitor, 45% chose an inhaled prostacyclin analog, 3.2% chose an endothelin receptor antagonist (ERA), and a single respondent did not choose a specific therapy. Of those clinicians who chose double agent therapy (*n* = 39), 55% chose the combination of a PDE5 inhibitor and an ERA, 32% chose the combination of a PDE5 inhibitor and an inhaled prostacyclin analog, and the remainder of clinicians chose various other combinations. There was no predominant combination for those who decided on triple agent therapy (*n* = 6), with clinicians selecting multiple combinations of PDE5 inhibitors, ERAs, oral/inhaled/IV/SQ prostacyclins, and riociguat. See Figure 5 for a visual representation of the individual answer choices for Case 3.

**Figure 6:**
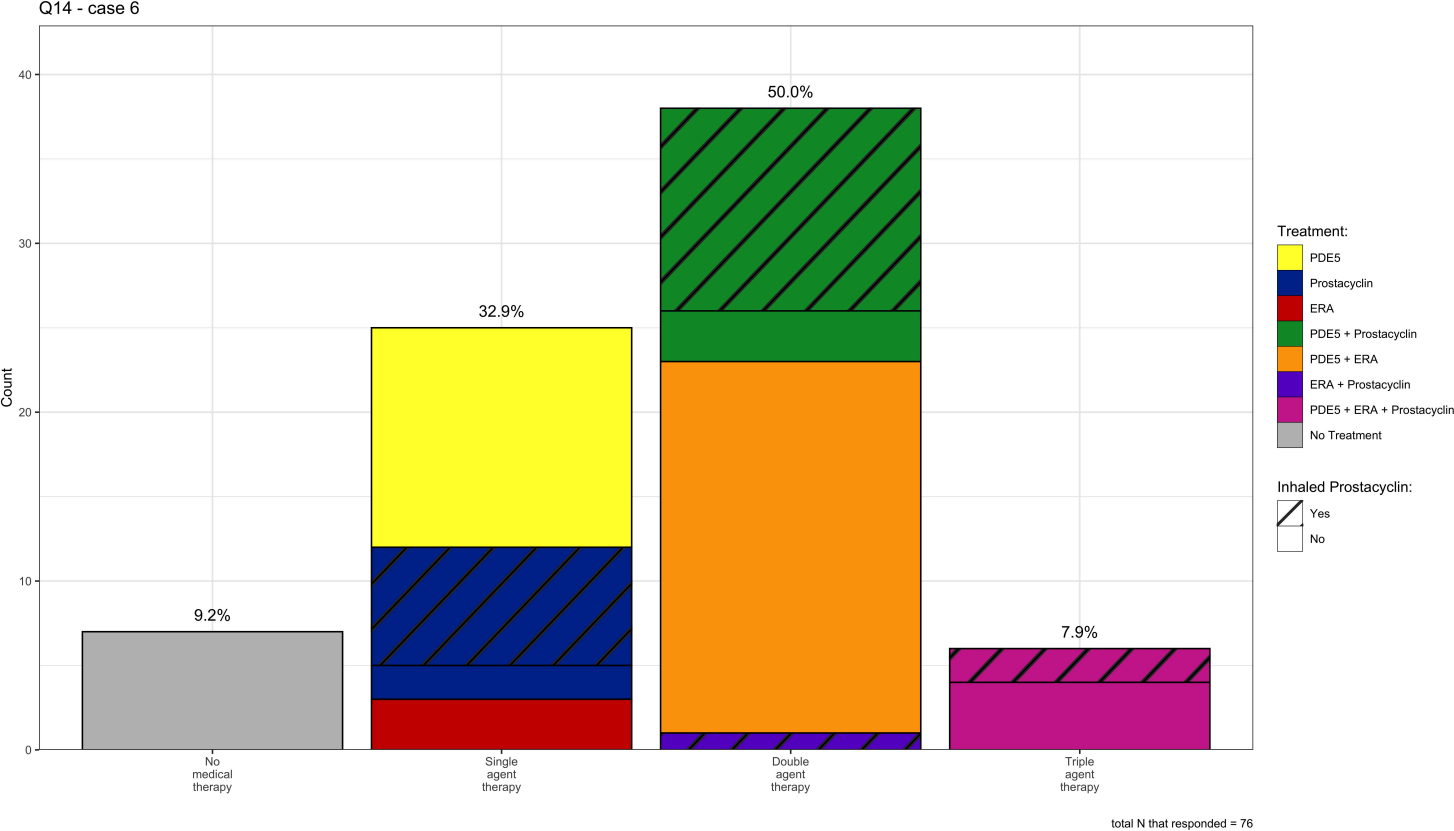
Individual medication choices for case 6 (mild COPD and severe PH), *N* = 76.

In Case 6 (mild COPD and severe PH), 33, 50, and 8% of clinicians chose single, double, and triple agent therapy, respectively. Of those clinicians who chose single-agent therapy (*n* = 25), 52% chose a PDE5 inhibitor, 28% chose an inhaled prostacyclin analog, and 12% chose an ERA. The remainder chose either an oral or IV/SQ prostacyclin. Of those clinicians who chose double agent therapy (*n* = 38), 58% chose the combination of PDE5 inhibitor and an ERA, 29% chose the combination of a PDE5 inhibitor and an inhaled prostacyclin analog, and the remainder of clinicians (*n* = 5) chose various other combinations. For those who decided on triple agent therapy, there was again not a predominant medication combination. See Figure 6 for a visual representation of the individual answer choices for Case 6.

In Case 4 (advanced IPF and severe PH), 37, 24, and 12% of clinicians chose single, double, and triple agent therapy, respectively. Of those clinicians who chose single-agent therapy (*n* = 30), 53% chose an inhaled prostacyclin analog, and 40% chose a PDE5 inhibitor. Of those clinicians who chose double agent therapy (*n* = 19), 74% chose the combination of a PDE5 inhibitor and an inhaled prostacyclin analog. Of those who decided on triple agent therapy (*n* = 10), there was no predominant combination.

In Case 7 (severe COPD and severe PH), 37, 24, and 11% of clinicians chose single, double and triple agent therapy, respectively. Of those clinicians who chose single-agent therapy (*n* = 28), 50% chose a PDE5 inhibitor, and 43% chose an inhaled prostacyclin analog. Of those clinicians who chose double agent therapy (*n* = 18), 39% chose the combination of PDE5 inhibitor and an inhaled prostacyclin analog, and 33% chose the combination of a PDE5 inhibitor and an IV/SQ prostacyclin analog. Of those clinicians who chose triple agent therapy, the predominant therapy choice was the combination of a PDE5 inhibitor, an ERA, and an IV/SQ prostacyclin analog. See the Appendix for all answer choices to every case.

## Discussion

In this study, we sought to characterize treatment preferences for PH-CLD. We hypothesized that there would be a wide variation in treatment practices among clinicians who treat this patient population. In cases with mild PH (mPAP <35 mmHg), clinicians predominantly chose no medical therapy, whereas in cases with severe PH (mPAP > 35 mmHg), clinicians were more likely to select PH therapy regardless of the severity of CLD. More than 90% of clinicians chose to treat patients with severe PH and mild CLD, while >70% chose to treat patients with severe PH and advanced CLD with PH-targeted therapies. There were clear patterns in selecting some form of medical treatment for the severe PH cases and no medical treatment for the mild PH cases. However, the Fleiss' kappa analysis of treatment strategy (i.e., 0 vs. 1 vs. 2 vs. 3 drug therapies) revealed poor overall agreement among clinicians regarding the initial treatment strategy. Our data shows that many clinicians would be inclined to treat PH-CLD with PH-targeted therapies even though these drugs, with the notable exception of inhaled treprostinil, are not approved for this indication.

Some observations in the choice of PH-targeted therapy are worth highlighting in light of reports questioning the safety of certain drugs and the approval of inhaled treprostinil almost a year after the survey was conducted. In the two cases with a “Group 1-like PH” phenotype, PDE5 inhibitors were the predominant choice, regardless of whether the clinician chose single, double or triple agent therapy. Several studies have been conducted to determine whether the treatment of COPD or ILD patients with PH with sildenafil offers some clinical benefit, but the data remains inconclusive (12–17). Interestingly, in case 3 (mild IPF and severe PH), many clinicians chose dual therapy with a PDE5 inhibitor and an ERA, despite evidence that the use of ERAs could worsen hypoxia without an increase in efficacy (18, 19). The ARTEMIS-IPF study was terminated prematurely because an interim analysis indicated that ambrisentan-treated patients with IPF were more likely to have disease progression and require hospitalizations due to respiratory decompensation (5). Of note, a parallel study to study ambrisentan in PH-CLD (ARTEMIS-PH) was also terminated by the results were never published. Regarding prostacyclins, there was a preference toward using inhaled prostacyclins as either monotherapy or in combination with other drugs (most often PDE5 inhibitors). This preference may have been motivated by multiple studies predating the recently completed INCREASE study suggesting that inhaled treprostinil can improve hemodynamics and functional capacity in PH-CLD (20–22). Of note, there was similarly poor agreement for the IPF vs. COPD cases and among both pulmonologists and cardiologists. The lack of agreement between disease subgroups or subspecialists likely reflects the wide variation in treatment practices, regardless of chronic lung disease type or training background.

Our study differs from the 2015 Trammel et al. (7) in several important ways. Besides capturing the present attitudes of clinicians familiar with the new 6th WSPH clinical definition of group 1 and group 3 PH (8), our survey is based on the use of hypothetical yet “real world” cases to assess the level of agreement amongst individual experts in their decision to treat or not to treat PH-IPF and PH-COPD. In contrast, the 2015 survey was designed to collect information from respondents on their diagnostic approach, their definition of “out of proportion” PH-CLD, and the percentage of non-group 1 patients treated with either single or combination PH-targeted therapy at their centers. This approach predated the current clinical classification of group 3 severity proposed by Nathan and colleagues at the 6th WSPH, which was used to design our cases (1). Also, it is worth noting that the 2015 Trammel study also included group 2 PH patients, another group for which no treatment guidelines currently exist. Despite their differences, our study complements the data captured in the 2015 survey by Trammel and should serve as a benchmark for future surveys on attitudes to PH-CLD treatment following the approval of inhaled treprostinil for PH-ILD.

There are several limitations to our study. We recognize that these are simplified cases and treatment plans that don't necessarily reflect complex real-world clinical scenarios and include common comorbidities and other treatment options such as pulmonary rehabilitation, palliative care, and lung transplant. We also acknowledge that the lack of details regarding the clinical context of individual cases may have led clinicians to select specific approaches that may not reflect their approach to management. Also, as this was a convenience sample, we were unable to calculate the response rate or characterize the non-respondents and likely selection bias. The respondents were overwhelmingly from the United States, and thus our survey results cannot be generalized to any other countries.

Despite these limitations, this survey data provides important information regarding current clinician treatment practices for the use of PH-targeted therapy in PH-CLD. We found that most clinicians chose to treat severe PH in patients with severe lung disease, despite a lack of treatment guidelines or approved medical therapies at the time of this survey. We strongly recommend that all clinicians closely adhere to published guidelines for patients with PH-CLD, which emphasize early referral to PH centers of comprehensive care and individualized treatment planning by experienced clinicians.

While our survey was administered before the results of the INCREASE trial had been published or the FDA had approved inhaled treprostinil, our data show that inhaled therapy is already part of many clinicians' treatment practices in PH-CLD. Use of inhaled prostanoids in PH in COPD patients also appears to be favored and reflects the interest in this treatment modality currently being tested in the ongoing PERFECT study evaluating the safety and efficacy of inhaled treprostinil in COPD patients (NCT03496623).

## Conclusion

Most clinicians who care for PH-CLD patients associated with IPF and COPD favor empirical therapy with PH targeted therapy despite the lack of consensus guidelines. The decision to offer treatment may be guided by the severity of the PH independent of the status of the CLD. Whereas, respondents favored the use of inhaled prostanoids, overall agreement regarding the use of one or multiple drug classes was low. Given the number of respondents that chose to use ERAs, it is vital to educate the medical community regarding the risks associated with PH-targeted therapies in PH-CLD. Thus, our results raise concern regarding the lack of proper guidance for the use of PH therapies in PH-CLD and stress the need for updated and perhaps better-informed guidelines.

## Data Availability Statement

The original contributions presented in the study are included in the article/Supplementary Material, further inquiries can be directed to the corresponding author/s.

## Guarantor Statement

VJP is the guarantor of the content of the manuscript.

## Author Contributions

VJP and RZ had full access to all the data in the study and take responsibility for its integrity and the data analysis and revised the manuscript for important intellectual content. All authors contributed to study design, data analysis, manuscript writing, and reviewed and revised the manuscript.

## Funding

This work was supported by an NIH R01 HL134776 and R01 HL139664 to VJP.

## Conflict of Interest

The authors declare that the research was conducted in the absence of any commercial or financial relationships that could be construed as a potential conflict of interest.

## Publisher's Note

All claims expressed in this article are solely those of the authors and do not necessarily represent those of their affiliated organizations, or those of the publisher, the editors and the reviewers. Any product that may be evaluated in this article, or claim that may be made by its manufacturer, is not guaranteed or endorsed by the publisher.

## Abbreviations

ACCP: American College of Chest Physicians
ATS: American Thoracic Society
CI: Cardiac index
CLD: Chronic lung disease
COPD: Chronic obstructive pulmonary disease
CTEPH: Chronic thromboembolic pulmonary hypertension
CTD: Connective tissue disease
DLCO: Diffusing capacity of the lungs for carbon monoxide
ERA: Endothelin receptor antagonist
FDA: Food and drug administration
FVC: Forced vital capacity
FEV1: Forced expiratory volume in 1 second
IPF: Idiopathic pulmonary fibrosis
HIV: Human Immunodeficiency Virus
ILD: Interstitial lung disease
IV: intravenous
VO2 max: maximal rate of oxygen consumption
mPAP: mean pulmonary artery pressure
NYHA: New York Heart Association
PDE5 inhibitor: Phosphodiesterase-5 inhibitor
PAH: Pulmonary arterial hypertension
PCWP: Pulmonary capillary wedge pressure
PFT: Pulmonary function test
PH: Pulmonary hypertension
PH-CLD: Pulmonary hypertension associated with chronic lung disease
PHCR: Pulmonary Hypertension Clinicians and Researchers
PVR: Pulmonary vascular resistance
RHC: Right heart catheterization
RV: Right ventricle
6MWD: Six minute walk distance
SC: subcutaneous
TR: tricuspid regurgitation
V/Q scan: ventilation/perfusion scan
WU: Wood units
WHO: World Health Organization
6^th^ WSPH: 6^th^ World Symposium on PH.
