# Supplementary material for: Prescription Patterns for Pulmonary Vasodilators in the Treatment of Pulmonary Hypertension Associated With Chronic Lung Diseases: Insights From a Clinician Survey
Source: Front Med (Lausanne). 2021 Dec 3;8:764815. doi: 10.3389/fmed.2021.764815 (PMC8677825; doi:10.3389/fmed.2021.764815)
Supplement: Supplementary file 1 [file Data_Sheet_1.PDF]

Demographics

Dear colleague,

Welcome to the Group 3 Pulmonary Hypertension Treatment Survey. The purpose of this survey is to help us to better understand treatment practices for patients with lung disease and pulmonary hypertension. We will collect basic demographic data to better understand practice patterns of different clinicians, institutions and regions. The data will be kept completely anonymous.

This survey includes some demographic questions and 7 hypothetical cases. **It should take you less than 10 minutes.**

Each case will give you several treatment options: no medical therapy or single/double/triple agent therapy. Depending which option you choose, you will be asked to select a specific combination of therapies. Please do not include any individually identifiable information in your answers.

You may have completed a prior version of this survey - please complete it again as we have updated the questions and cases.

Participant’s rights questions, contact 1-866-680-2906.

Abbreviations: Idiopathic pulmonary fibrosis (IPF); New York Heart Association (NYHA); forced vital capacity (FVC); forced expiratory volume in 1 second (FEV1); diffusing capacity of the lungs for carbon monoxide (DLCO); mean pulmonary artery pressure (mPAP); pulmonary vascular resistance (PVR); Wood Units (WU); pulmonary capillary wedge pressure (PCWP); cardiac index (CI); six minute walk distance (6MWD); maximal rate of oxygen consumption (VO2 max); ventilation perfusion scan (V/Q scan); human immunodeficiency virus (HIV); intravenous (IV); subcutaneous (SC); right ventricle (RV); tricuspid regurgitation (TR); chronic obstructive pulmonary disease (COPD)

Block 1

Which best describes your title?

- ◯ Attending physician / consultant

- Physician-in-training
- Nurse Practitioner
- Physician Assistant
- Other

Please describe your title

Block 2

Which field best describes your training background?

- Pulmonary medicine
- Cardiovascular medicine
- Rheumatology
- Other

Please describe your training background

Block 3

How many years have you been practicing after completion of your training program(s)?

Block 4

Do you practice in the United States?

- Yes
- No

Which best describes the type of institution in which you practice primarily?

- Pulmonary Hypertension Center of Comprehensive Care
- Pulmonary Hypertension Regional Clinical Program
- Academic Center without PHA designation
- Community Practice without PHA designation
- Other

Please enter a brief description of your primary practice institution

Block 5

Please enter the first three digits of the zip code in which you practice (this will be kept completely anonymous)

Block 6

What percentage of your time do you spend practicing clinical medicine (i.e. not administration, research, etc.)?

0 10 20 30 40 50 60 70 80 90 100

Block 7

What percentage of your clinical practice is treating pulmonary hypertension patients?

0 10 20 30 40 50 60 70 80 90 100

Block 8

Approximately how many unique patients with group 3 pulmonary hypertension have you seen in the last year in your practice?

Case 1 Block

Case 2 Block

Demographics	
Age	58
Sex	Male
Disease	Advanced IPF with extensive parenchymal involvement
WHO Functional Class	III
**PFTs**	
FEV1/FVC ratio	0.78
FVC (% predicted)	50%
DLCO (% predicted)	47%
Echo	normal biventricular size and function, normal valves

Right Heart Catheterization	
mPAP	31 mmHg
PVR	4.1 WU
PCWP	10 mmHg
CI	2.7 L/min/m^2
**Other Testing**	
6MWD	200 meters
VO2 Max	13 ml/min/kg
V/Q Scan	Low probability for PE
Autoimmune serologies	Negative
Kidney and liver function	Normal
HIV	Negative

What would be your initial therapeutic approach to treating the pulmonary hypertension in this patient?

- No medical therapy
- Single agent therapy
- Double agent therapy
- Triple agent therapy

Which single agent therapy would you choose?

- PDE5 inhibitor
- Endothelin receptor antagonist
- Oral prostacyclin analogue or receptor agonist
- Inhaled prostacyclin analogue
- IV/SQ prostacyclin analogue
- Riociguat

Which double agent therapy combination would you choose?

- PDE5 inhibitor + endothelin receptor antagonist
- PDE5 inhibitor + oral prostacyclin analogue or receptor agonist
- PDE5 inhibitor + inhaled prostacyclin analogue
- PDE5 inhibitor + IV/SQ prostacyclin analogue
- Endothelin receptor antagonist + oral prostacyclin analogue or receptor agonist
- Endothelin receptor antagonist + inhaled prostacyclin analogue
- Endothelin receptor antagonist + IV/SQ prostacyclin analogue
- Endothelin receptor antagonist + riociguat
- Oral prostacyclin analogue or receptor agonist + riociguat
- Inhaled prostacyclin analogue + riociguat
- IV/SQ prostacyclin analogue + riociguat

Which triple agent therapy combination would you choose?

- ◯ PDE5 inhibitor + endothelin receptor antagonist + oral prostacyclin analogue or receptor agonist
- ◯ PDE5 inhibitor + endothelin receptor antagonist + inhaled prostacyclin analogue
- ◯ PDE5 inhibitor + endothelin receptor antagonist + IV/SQ prostacyclin analogue
- ◯ Riociguat + endothelin receptor antagonist + oral prostacyclin analogue or receptor agonist
- ◯ Riociguat + endothelin receptor antagonist + inhaled prostacyclin analogue
- ◯ Riociguat + endothelin receptor antagonist + IV/SQ prostacyclin analogue

Case 3 Block

Demographics	
Age	61
Sex	Female
Disease	Mild IPF with limited parenchymal involvement
WHO Functional Class	III
**PFTs**	
FEV1/FVC ratio	0.73
FVC (% predicted)	85%
DLCO (% predicted)	35%
Echo	moderate RV dilation with reduced systolic function, normal left sided size and function, and moderate TR

Right Heart Catheterization	
mPAP	39 mmHg
PVR	8.75 WU
PCWP	11 mmHg
CI	2.0 L/min/m^2
**Other Testing**	
6MWD	300 meters
VO2 Max	14 ml/min/kg
V/Q Scan	Low probability for PE
Autoimmune serologies	Negative
Kidney and liver function	Normal
HIV	Negative

What would be your initial therapeutic approach
to treating the pulmonary hypertension in this patient?

- ◯ No medical therapy

- ◯ Single agent therapy
- ◯ Double agent therapy
- ◯ Triple agent therapy

Which single agent therapy would you choose?

- ◯ PDE5 inhibitor
- ◯ Endothelin receptor antagonist
- ◯ Oral prostacyclin analogue or receptor agonist
- ◯ Inhaled prostacyclin analogue
- ◯ IV/SQ prostacyclin analogue
- ◯ Riociguat

Which double agent therapy combination would you choose?

- ◯ PDE5 inhibitor + endothelin receptor antagonist
- ◯ PDE5 inhibitor + oral prostacyclin analogue or receptor agonist
- ◯ PDE5 inhibitor + inhaled prostacyclin analogue
- ◯ PDE5 inhibitor + IV/SQ prostacyclin analogue
- ◯ Endothelin receptor antagonist + oral prostacyclin analogue or receptor agonist
- ◯ Endothelin receptor antagonist + inhaled prostacyclin analogue
- ◯ Endothelin receptor antagonist + IV/SQ prostacyclin analogue
- ◯ Endothelin receptor antagonist + riociguat

- Oral prostacyclin analogue or receptor agonist + riociguat
- Inhaled prostacyclin analogue + riociguat
- IV/SQ prostacyclin analogue + riociguat

Which triple agent therapy combination would you choose?

- PDE5 inhibitor + endothelin receptor antagonist + oral prostacyclin analogue or receptor agonist
- PDE5 inhibitor + endothelin receptor antagonist + inhaled prostacyclin analogue
- PDE5 inhibitor + endothelin receptor antagonist + IV/SQ prostacyclin analogue
- Riociguat + endothelin receptor antagonist + oral prostacyclin analogue or receptor agonist
- Riociguat + endothelin receptor antagonist + inhaled prostacyclin analogue
- Riociguat + endothelin receptor antagonist + IV/SQ prostacyclin analogue

Case 4 Block

Demographics	
Age	57
Sex	Male
Disease	Advanced IPF with extensive parenchymal involvement
WHO Functional Class	III
**PFTs**	
FEV1/FVC ratio	0.74
FVC (% predicted)	45%
DLCO (% predicted)	22%
Echo	moderate RV dilation with reduced systolic function, severe TR, and normal left sided size and function

Right Heart Catheterization	
mPAP	46 mmHg
PVR	10.3 WU
PCWP	9 mmHg
CI	1.9 L/min/m^2
**Other Testing**	
6MWD	150 meters
VO2 Max	9 ml/min/kg
V/Q Scan	Low probability for PE
Autoimmune serologies	Negative
Kidney and liver function	Normal
HIV	Negative

What would be your initial therapeutic approach to treating the pulmonary hypertension in this patient?

- ○ No medical therapy
- ○ Single agent therapy
- ○ Double agent therapy
- ○ Triple agent therapy

Which single agent therapy would you choose?

- ○ PDE5 inhibitor
- ○ Endothelin receptor antagonist
- ○ Oral prostacyclin analogue or receptor agonist
- ○ Inhaled prostacyclin analogue
- ○ IV/SQ prostacyclin analogue
- ○ Riociguat

Which double agent therapy combination would you choose?

- ◯ PDE5 inhibitor + endothelin receptor antagonist
- ◯ PDE5 inhibitor + oral prostacyclin analogue or receptor agonist
- ◯ PDE5 inhibitor + inhaled prostacyclin analogue
- ◯ PDE5 inhibitor + IV/SQ prostacyclin analogue
- ◯ Endothelin receptor antagonist + oral prostacyclin analogue or receptor agonist
- ◯ Endothelin receptor antagonist + inhaled prostacyclin analogue
- ◯ Endothelin receptor antagonist + IV/SQ prostacyclin analogue
- ◯ Endothelin receptor antagonist + riociguat
- ◯ Oral prostacyclin analogue or receptor agonist + riociguat
- ◯ Inhaled prostacyclin analogue + riociguat
- ◯ IV/SQ prostacyclin analogue + riociguat

Which triple agent therapy combination would you choose?

- ◯ PDE5 inhibitor + endothelin receptor antagonist + oral prostacyclin analogue or receptor agonist
- ◯ PDE5 inhibitor + endothelin receptor antagonist + inhaled prostacyclin analogue
- ◯ PDE5 inhibitor + endothelin receptor antagonist + IV/SQ prostacyclin analogue
- ◯ Riociguat + endothelin receptor antagonist + oral prostacyclin analogue or receptor agonist

- ◯ Riociguat + endothelin receptor antagonist + inhaled prostacyclin analogue
- ◯ Riociguat + endothelin receptor antagonist + IV/SQ prostacyclin analogue

Case 5 Block

Demographics	
Age	62
Sex	Female
Disease	Mild COPD
WHO Functional Class	II

PFTs	
FEV1/FVC ratio	0.62
FEV1 (% predicted)	59%
FVC (% predicted)	81%
DLCO (% predicted)	65%
Echo	normal biventricular size and function, normal valves

Right Heart Catheterization	
mPAP	31 mmHg
PVR	5.0 WU
PCWP	8 mmHg
CI	2.9 L/min/m^2

Other Testing	
6MWD	550 meters
VO2 Max	18 ml/min/kg
V/Q Scan	Low probability for PE
Autoimmune serologies	Negative
Kidney and liver function	Normal
HIV	Negative

What would be your initial therapeutic approach to treating the pulmonary hypertension in this patient?

- ◯ No medical therapy
- ◯ Single agent therapy
- ◯ Double agent therapy
- ◯ Triple agent therapy

Which single agent therapy would you choose?

- PDE5 inhibitor
- Endothelin receptor antagonist
- Oral prostacyclin analogue or receptor agonist
- Inhaled prostacyclin analogue
- IV/SQ prostacyclin analogue
- Riociguat

Which double agent therapy combination would you choose?

- PDE5 inhibitor + endothelin receptor antagonist
- PDE5 inhibitor + oral prostacyclin analogue or receptor agonist
- PDE5 inhibitor + inhaled prostacyclin analogue
- PDE5 inhibitor + IV/SQ prostacyclin analogue
- Endothelin receptor antagonist + oral prostacyclin analogue or receptor agonist
- Endothelin receptor antagonist + inhaled prostacyclin analogue
- Endothelin receptor antagonist + IV/SQ prostacyclin analogue
- Endothelin receptor antagonist + riociguat
- Oral prostacyclin analogue or receptor agonist + riociguat
- Inhaled prostacyclin analogue + riociguat
- IV/SQ prostacyclin analogue + riociguat

Which triple agent therapy combination would you choose?

- PDE5 inhibitor + endothelin receptor antagonist + oral prostacyclin analogue or receptor agonist
- PDE5 inhibitor + endothelin receptor antagonist + inhaled prostacyclin analogue
- PDE5 inhibitor + endothelin receptor antagonist + IV/SQ prostacyclin analogue
- Riociguat + endothelin receptor antagonist + oral prostacyclin analogue or receptor agonist
- Riociguat + endothelin receptor antagonist + inhaled prostacyclin analogue
- Riociguat + endothelin receptor antagonist + IV/SQ prostacyclin analogue

Case 6 Block

Demographics	
Age	53
Sex	Male
Disease	Mild COPD
WHO Functional Class	III
**PFTs**	
FEV1/FVC ratio	0.59
FEV1 (% predicted)	63%
FVC (% predicted)	91%
DLCO (% predicted)	38%
Echo	moderate RV dilation with reduced systolic function, normal left sided size and function, and moderate TR

Right Heart Catheterization	
mPAP	45 mmHg
PVR	9.2 WU
PCWP	10 mmHg
CI	2.0 L/min/m^2
**Other Testing**	
6MWD	275 meters
VO2 Max	14 ml/min/kg
V/Q Scan	Low probability for PE
Autoimmune serologies	Negative
Kidney and liver function	Normal
HIV	Negative

What would be your initial therapeutic approach to treating the pulmonary hypertension in this patient?

- No medical therapy

- ◯ Single agent therapy
- ◯ Double agent therapy
- ◯ Triple agent therapy

Which single agent therapy would you choose?

- ◯ PDE5 inhibitor
- ◯ Endothelin receptor antagonist
- ◯ Oral prostacyclin analogue or receptor agonist
- ◯ Inhaled prostacyclin analogue
- ◯ IV/SQ prostacyclin analogue
- ◯ Riociguat

Which double agent therapy combination would you choose?

- ◯ PDE5 inhibitor + endothelin receptor antagonist
- ◯ PDE5 inhibitor + oral prostacyclin analogue or receptor agonist
- ◯ PDE5 inhibitor + inhaled prostacyclin analogue
- ◯ PDE5 inhibitor + IV/SQ prostacyclin analogue
- ◯ Endothelin receptor antagonist + oral prostacyclin analogue or receptor agonist
- ◯ Endothelin receptor antagonist + inhaled prostacyclin analogue
- ◯ Endothelin receptor antagonist + IV/SQ prostacyclin analogue
- ◯ Endothelin receptor antagonist + riociguat

- Oral prostacyclin analogue or receptor agonist + riociguat
- Inhaled prostacyclin analogue + riociguat
- IV/SQ prostacyclin analogue + riociguat

Which triple agent therapy combination would you choose?

- PDE5 inhibitor + endothelin receptor antagonist + oral prostacyclin analogue or receptor agonist
- PDE5 inhibitor + endothelin receptor antagonist + inhaled prostacyclin analogue
- PDE5 inhibitor + endothelin receptor antagonist + IV/SQ prostacyclin analogue
- Riociguat + endothelin receptor antagonist + oral prostacyclin analogue or receptor agonist
- Riociguat + endothelin receptor antagonist + inhaled prostacyclin analogue
- Riociguat + endothelin receptor antagonist + IV/SQ prostacyclin analogue

Case 7 Block

Demographics		Right Heart Catheterization	
Age	66	mPAP	59 mmHg
Sex	Female	PVR	16.1 WU
Disease	Severe COPD	PCWP	10 mmHg
WHO Functional Class	III	CI	1.9 L/min/m^2
**PFTs**		**Other Testing**	
FEV1/FVC ratio	0.42	6MWD	200 meters
FEV1 (% predicted)	29%	VO2 Max	9 ml/min/kg
FVC (% predicted)	71%	V/Q Scan	Low probability for PE
DLCO (% predicted)	31%	Autoimmune serologies	Negative
Echo	severe RV dilation with reduced systolic function, severe TR, and normal left sided size and function	Kidney and liver function	Normal
		HIV	Negative

What would be your initial therapeutic approach to treating the pulmonary hypertension in this patient?

- ○ No medical therapy
- ○ Single agent therapy
- ○ Double agent therapy
- ○ Triple agent therapy

Which single agent therapy would you choose?

- ○ PDE5 inhibitor
- ○ Endothelin receptor antagonist
- ○ Oral prostacyclin analogue or receptor agonist
- ○ Inhaled prostacyclin analogue
- ○ IV/SQ prostacyclin analogue
- ○ Riociguat

Which double agent therapy combination would you choose?

- ◯ PDE5 inhibitor + endothelin receptor antagonist
- ◯ PDE5 inhibitor + oral prostacyclin analogue or receptor agonist
- ◯ PDE5 inhibitor + inhaled prostacyclin analogue
- ◯ PDE5 inhibitor + IV/SQ prostacyclin analogue
- ◯ Endothelin receptor antagonist + oral prostacyclin analogue or receptor agonist
- ◯ Endothelin receptor antagonist + inhaled prostacyclin analogue
- ◯ Endothelin receptor antagonist + IV/SQ prostacyclin analogue
- ◯ Endothelin receptor antagonist + riociguat
- ◯ Oral prostacyclin analogue or receptor agonist + riociguat
- ◯ Inhaled prostacyclin analogue + riociguat
- ◯ IV/SQ prostacyclin analogue + riociguat

Which triple agent therapy combination would you choose?

- ◯ PDE5 inhibitor + endothelin receptor antagonist + oral prostacyclin analogue or receptor agonist
- ◯ PDE5 inhibitor + endothelin receptor antagonist + inhaled prostacyclin analogue
- ◯ PDE5 inhibitor + endothelin receptor antagonist + IV/SQ prostacyclin analogue
- ◯ Riociguat + endothelin receptor antagonist + oral prostacyclin analogue or receptor agonist

- ◯ Riociguat + endothelin receptor antagonist + inhaled prostacyclin analogue
- ◯ Riociguat + endothelin receptor antagonist + IV/SQ prostacyclin analogue

Powered by Qualtrics
