# Supplementary material for: Prescription Patterns for Pulmonary Vasodilators in the Treatment of Pulmonary Hypertension Associated With Chronic Lung Diseases: Insights From a Clinician Survey
Source: Front Med (Lausanne). 2021 Dec 3;8:764815. doi: 10.3389/fmed.2021.764815 (PMC8677825; doi:10.3389/fmed.2021.764815)

Group 3 PH Survey

This is an R Markdown Notebook. When you execute code within the notebook, the results appear beneath the code.

Try executing this chunk by clicking the *Run* button within the chunk or by placing your cursor inside it and pressing *Cmd+Shift+Enter*.

```
## Loading required package: lpSolve

## Loading required package: psych

##
## Attaching package: 'psych'

## The following objects are masked from 'package:ggplot2':
##
##     %+%, alpha

## Loading required package: likert

## Loading required package: xtable

## Loading required package: dplyr

##
## Attaching package: 'dplyr'

## The following object is masked from 'package:likert':
##
##     recode

## The following objects are masked from 'package:stats':
##
##     filter, lag

## The following objects are masked from 'package:base':
##
##     intersect, setdiff, setequal, union

## Loading required package: tableone

##
## Attaching package: 'ggpattern'

## The following object is masked from 'package:psych':
##
##     alpha

## The following objects are masked from 'package:ggplot2':
##
##     flip_data, flipped_names, gg_dep, has_flipped_aes, remove_missing,
##     should_stop, waiver
```

```
## table 1
print(tab1, nonnormal = c("Q3:years_practice", "Q6.1:percent_practice", "Q7.1:percent_PH", "Q8:num_uniq
```

```
##
##                                                          Overall
##   n                                                         87
##   Q1:title (%)
##      Attending physician / consultant                       84 (96.6)
##      Physician-in-training                                   1 ( 1.1)
##      Nurse Practitioner                                      2 ( 2.3)
##   Q2:training (%)
##      Pulmonary medicine                                     60 (69.0)
##      Cardiovascular medicine                                26 (29.9)
##      Other                                                   1 ( 1.1)
##   Q3:years_practice (median [IQR])                      12.00 [6.00, 20.00]
##   Q4:US = Yes (%)                                           80 (92.0)
##   Q4.1:institution_type (%)
##      Pulmonary Hypertension Center of Comprehensive Care    39 (48.8)
##      Pulmonary Hypertension Regional Clinical Program        4 ( 5.0)
##      Academic Center without PHA designation                28 (35.0)
##      Community Practice without PHA designation              7 ( 8.8)
##      Other                                                   2 ( 2.5)
##   Q6.1:percent_practice (median [IQR])                  75.00 [60.00, 81.00]
##   Q7.1:percent_PH (median [IQR])                        50.00 [30.00, 76.00]
##   Q8:num_uniquepatients (median [IQR])                  30.00 [20.00, 50.00]
##   State (%)
##      AZ                                                      2 ( 2.5)
##      CA                                                      6 ( 7.6)
##      CO                                                      3 ( 3.8)
##      CT                                                      2 ( 2.5)
##      DC                                                      1 ( 1.3)
##      FL                                                      9 (11.4)
##      HI                                                      1 ( 1.3)
##      IL                                                      1 ( 1.3)
##      IN                                                      2 ( 2.5)
##      KY                                                      2 ( 2.5)
##      LA                                                      1 ( 1.3)
##      MA                                                      4 ( 5.1)
##      MD                                                      2 ( 2.5)
##      MI                                                      2 ( 2.5)
##      MN                                                      3 ( 3.8)
##      MO                                                      1 ( 1.3)
##      NC                                                      3 ( 3.8)
##      NM                                                      2 ( 2.5)
##      NY                                                      3 ( 3.8)
##      OH                                                      4 ( 5.1)
##      PA                                                      7 ( 8.9)
##      RI                                                      1 ( 1.3)
##      SC                                                      1 ( 1.3)
##      TN                                                      3 ( 3.8)
##      TX                                                      5 ( 6.3)
##      VA                                                      4 ( 5.1)
##      VT                                                      1 ( 1.3)
##      WI                                                      2 ( 2.5)
##      WV                                                      1 ( 1.3)
```

Q9 − case 1

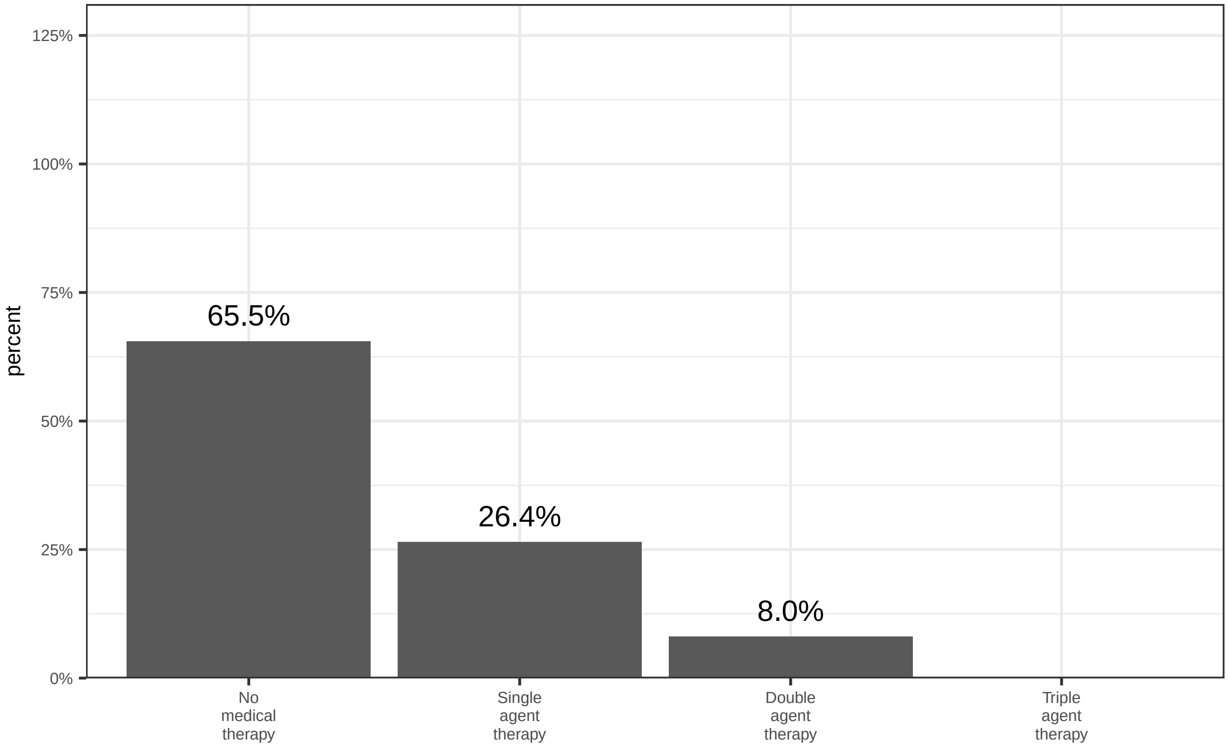

total N that responded = 87

Which single agent therapy would you choose?

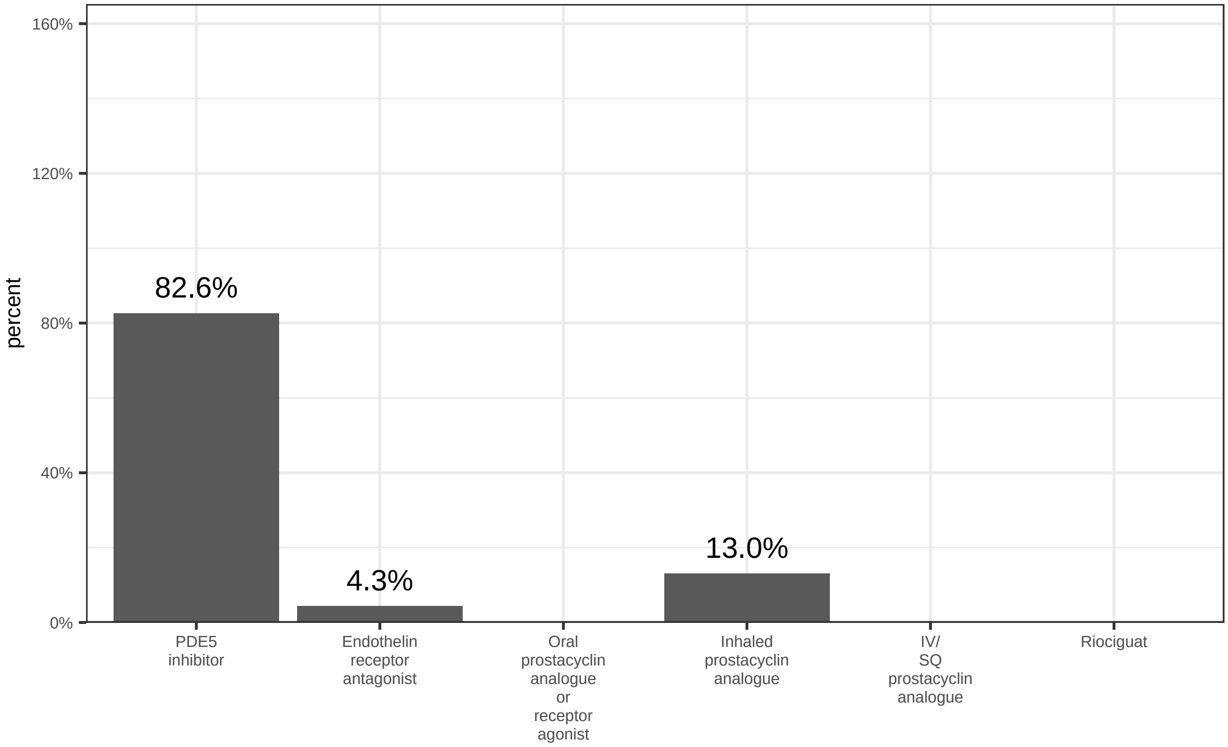

total N that responded = 23

Which double agent therapy combination would you choose?

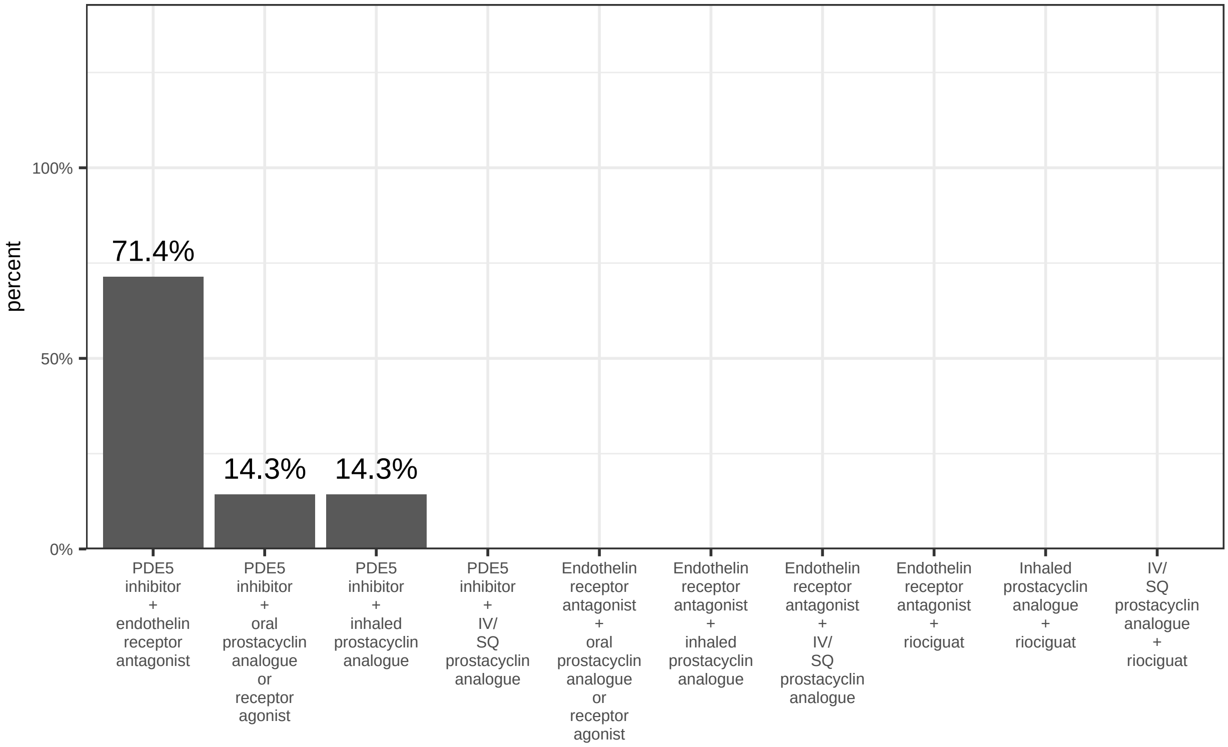

total N that responded = 7

Q10 − case 2

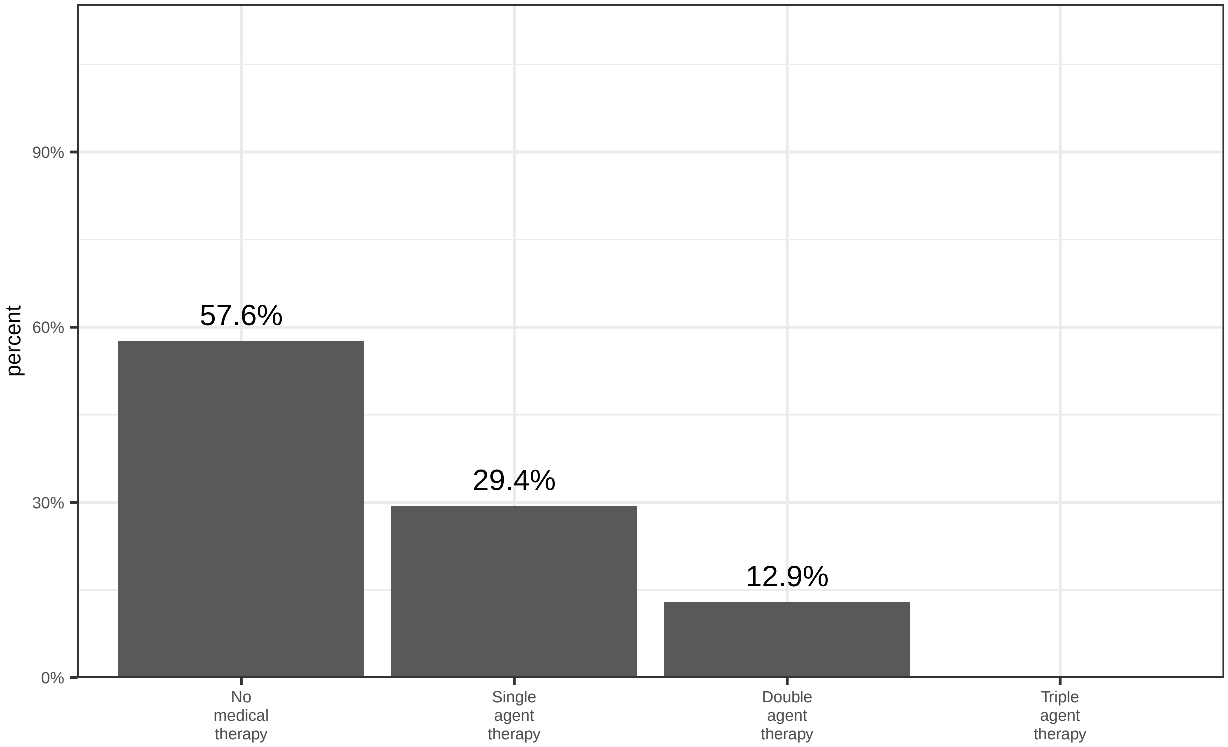

total N that responded = 85

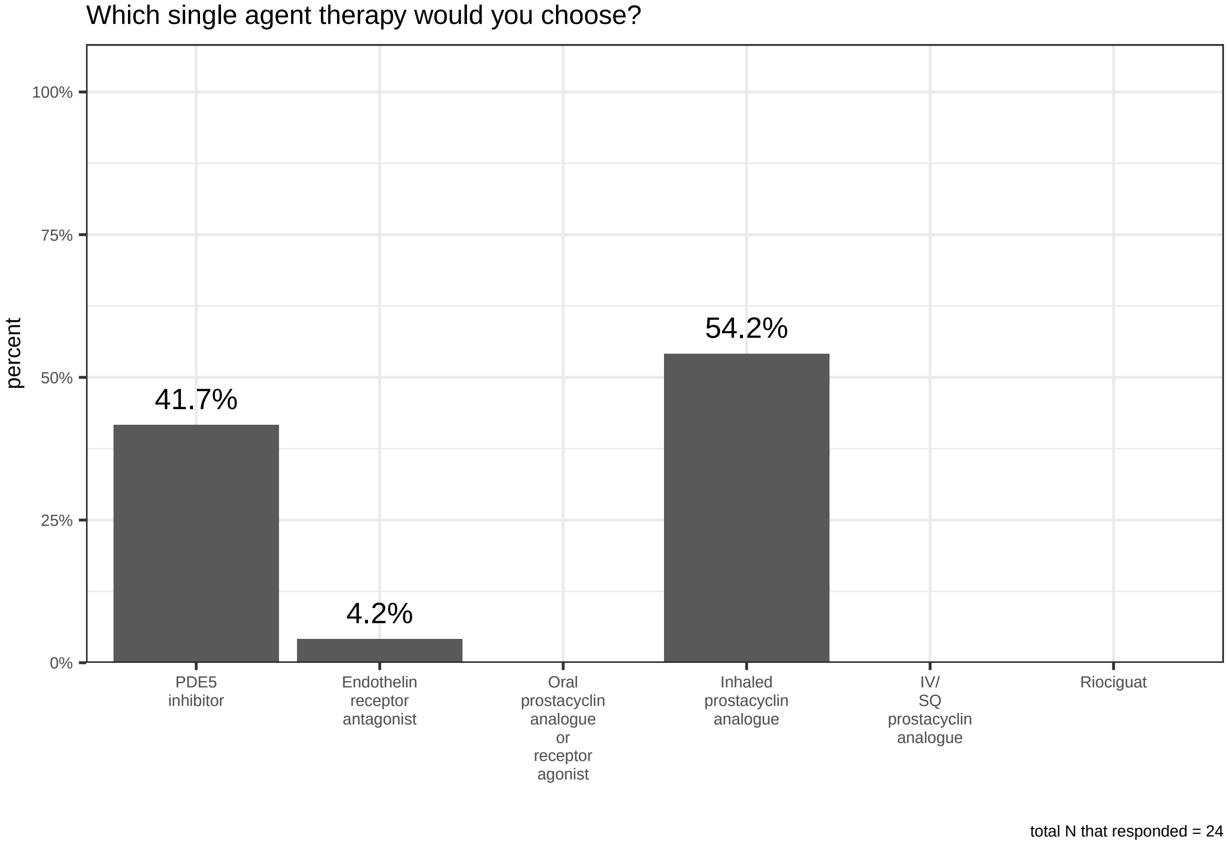
Which single agent therapy would you choose?
percent
100%
75%
50%
25%
0%
41.7%
4.2%
54.2%
PDE5 inhibitor
Endothelin receptor antagonist
Oral prostacyclin analogue or receptor agonist
Inhaled prostacyclin analogue
IV/ SQ prostacyclin analogue
Riociguat
total N that responded = 24

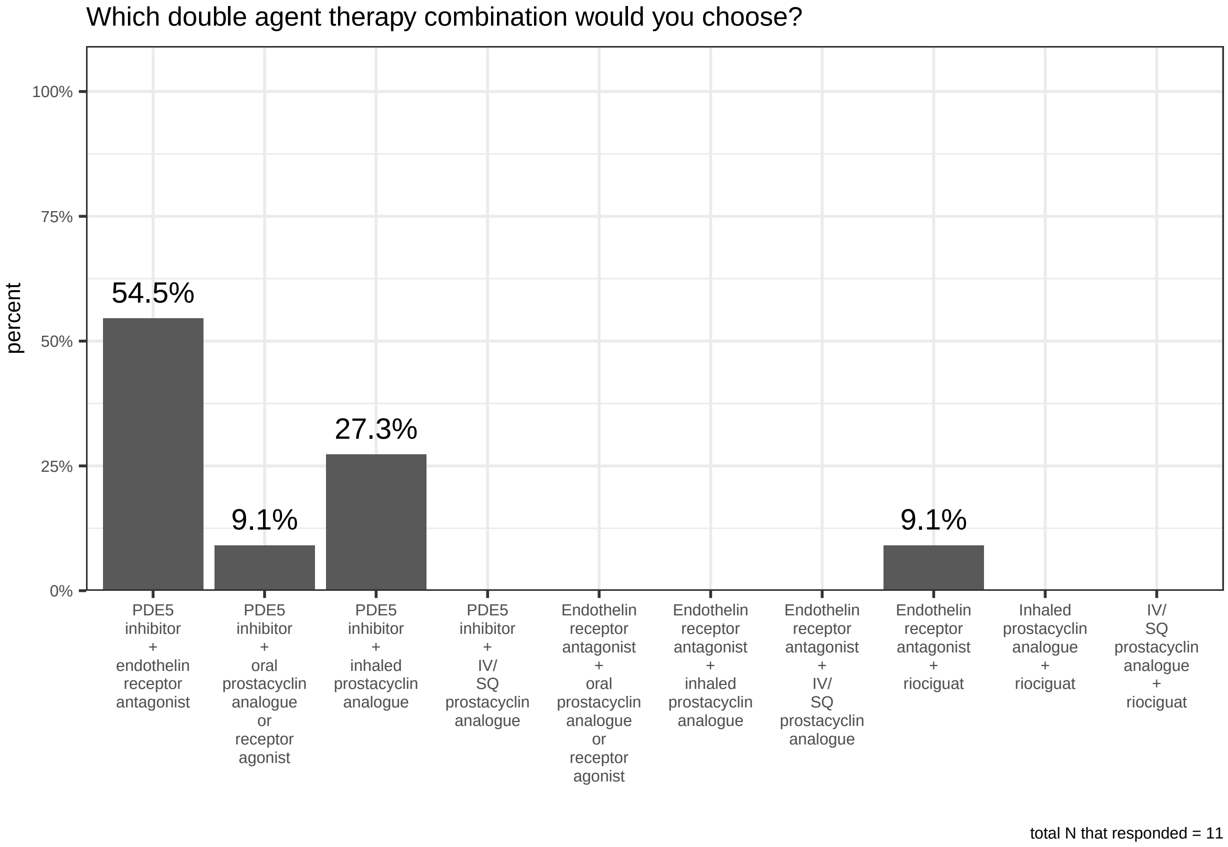
Which double agent therapy combination would you choose?
percent
100%
75%
50%
25%
0%
54.5%
9.1%
27.3%
9.1%
PDE5 inhibitor + endothelin receptor antagonist
PDE5 inhibitor + oral prostacyclin analogue or receptor agonist
PDE5 inhibitor + inhaled prostacyclin analogue
PDE5 inhibitor + IV/ SQ prostacyclin analogue
Endothelin receptor antagonist + oral prostacyclin analogue or receptor agonist
Endothelin receptor antagonist + inhaled prostacyclin analogue
Endothelin receptor antagonist + IV/ SQ prostacyclin analogue
Endothelin receptor antagonist + riociguat
Inhaled prostacyclin analogue + riociguat
IV/ SQ prostacyclin analogue + riociguat
total N that responded = 11

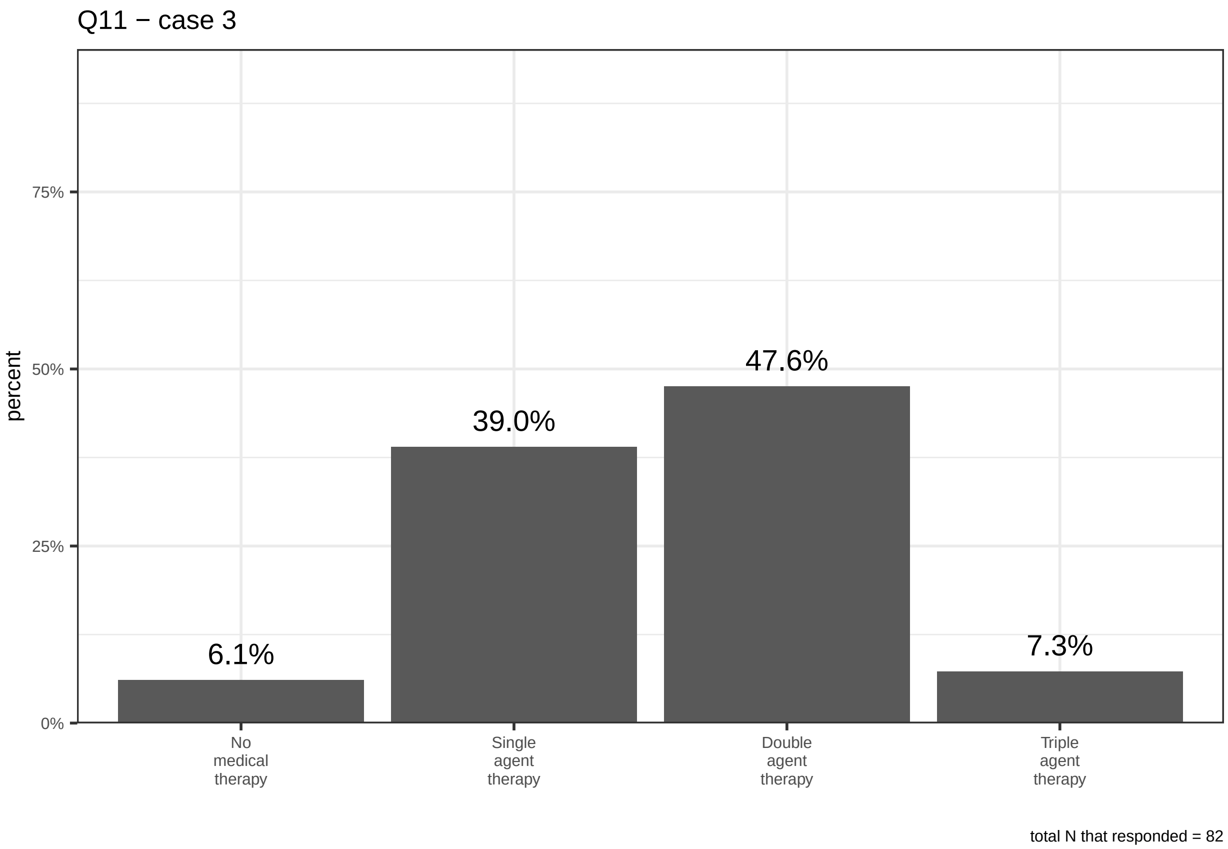
Q11 − case 3
percent
75%
50%
25%
0%
6.1%
39.0%
47.6%
7.3%
No medical therapy
Single agent therapy
Double agent therapy
Triple agent therapy
total N that responded = 82

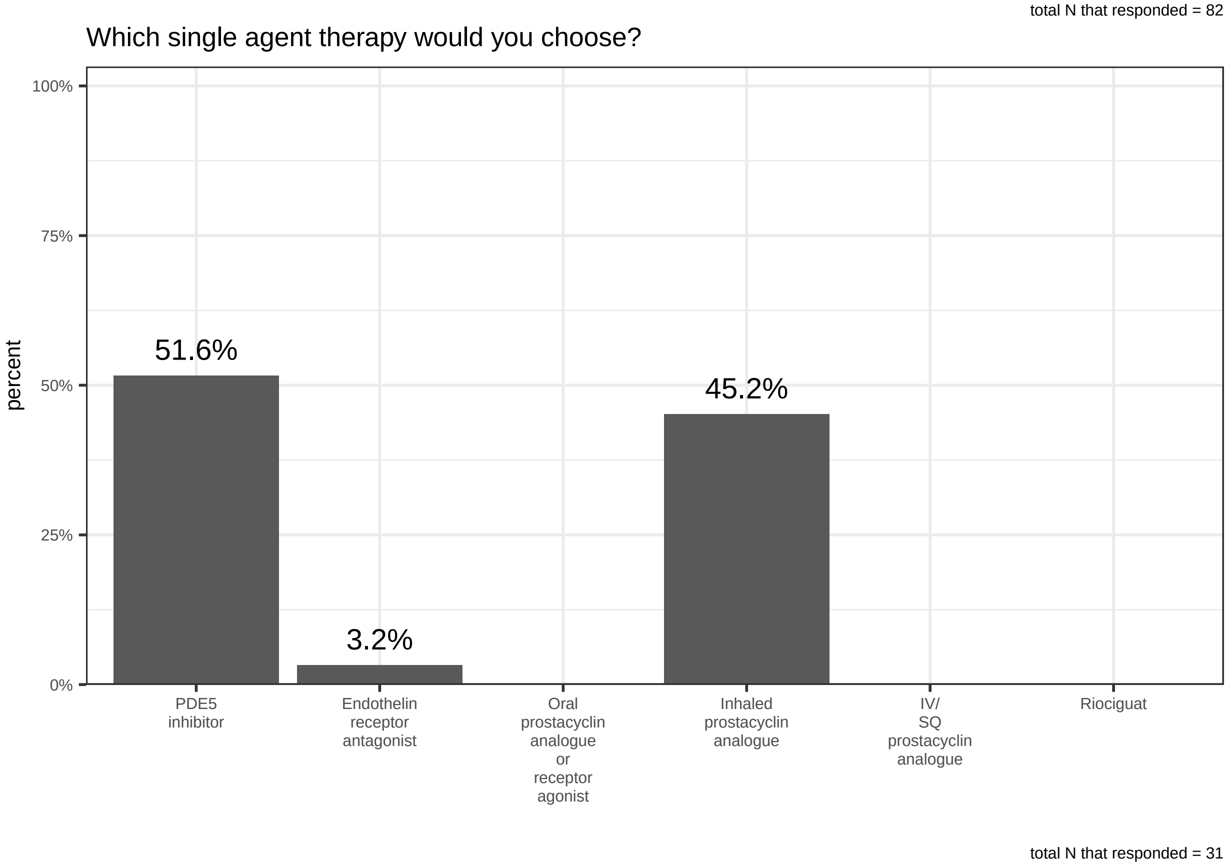
Which single agent therapy would you choose?
percent
100%
75%
50%
25%
0%
51.6%
3.2%
45.2%
PDE5 inhibitor
Endothelin receptor antagonist
Oral prostacyclin analogue or receptor agonist
Inhaled prostacyclin analogue
IV/ SQ prostacyclin analogue
Riociguat
total N that responded = 31

Which double agent therapy combination would you choose?

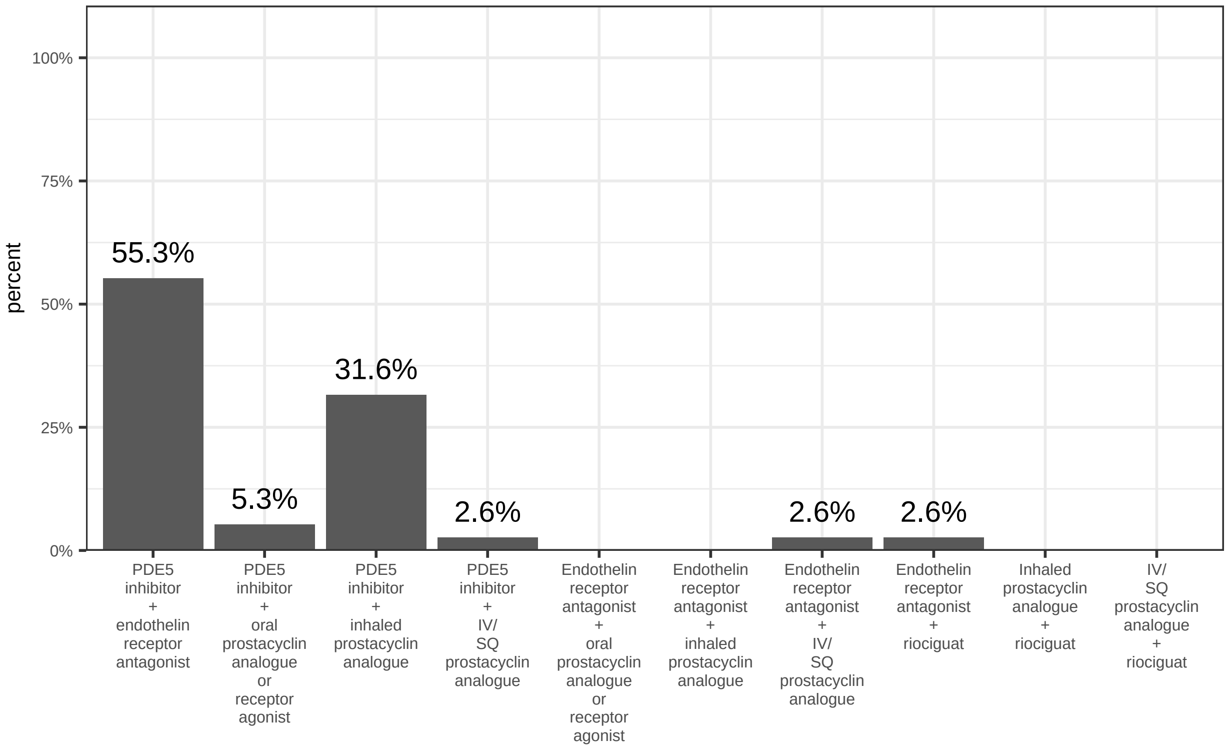

total N that responded = 38

Which triple agent therapy combination would you choose?

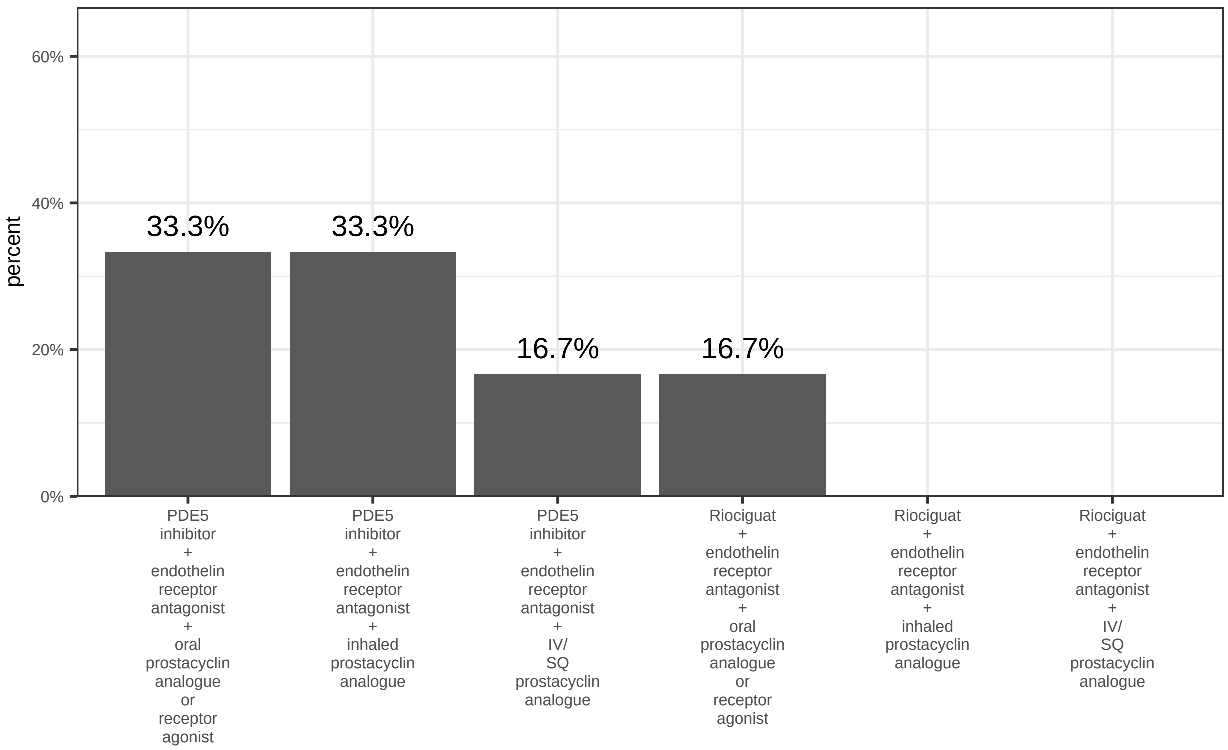

total N that responded = 6

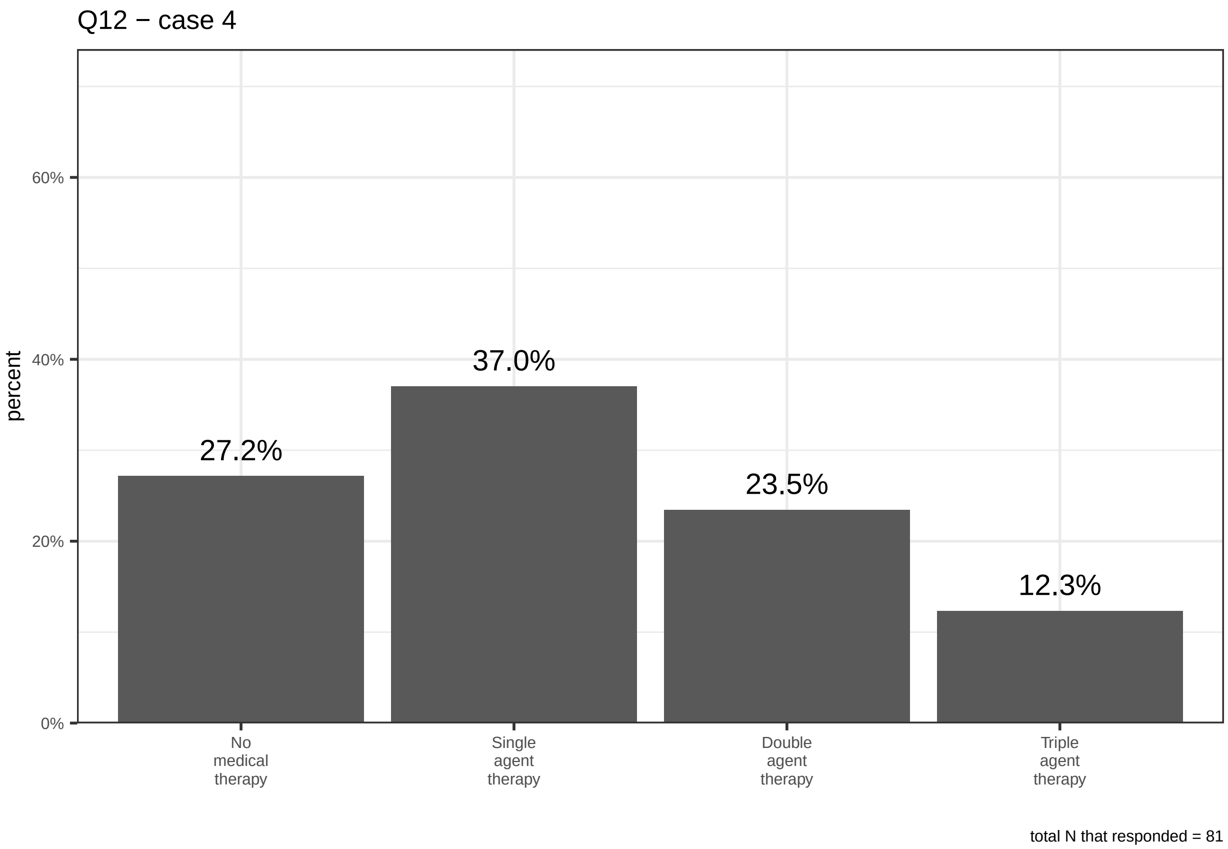
Q12 − case 4
percent
60%
40%
20%
0%
37.0%
27.2%
23.5%
12.3%
No medical therapy
Single agent therapy
Double agent therapy
Triple agent therapy
total N that responded = 81

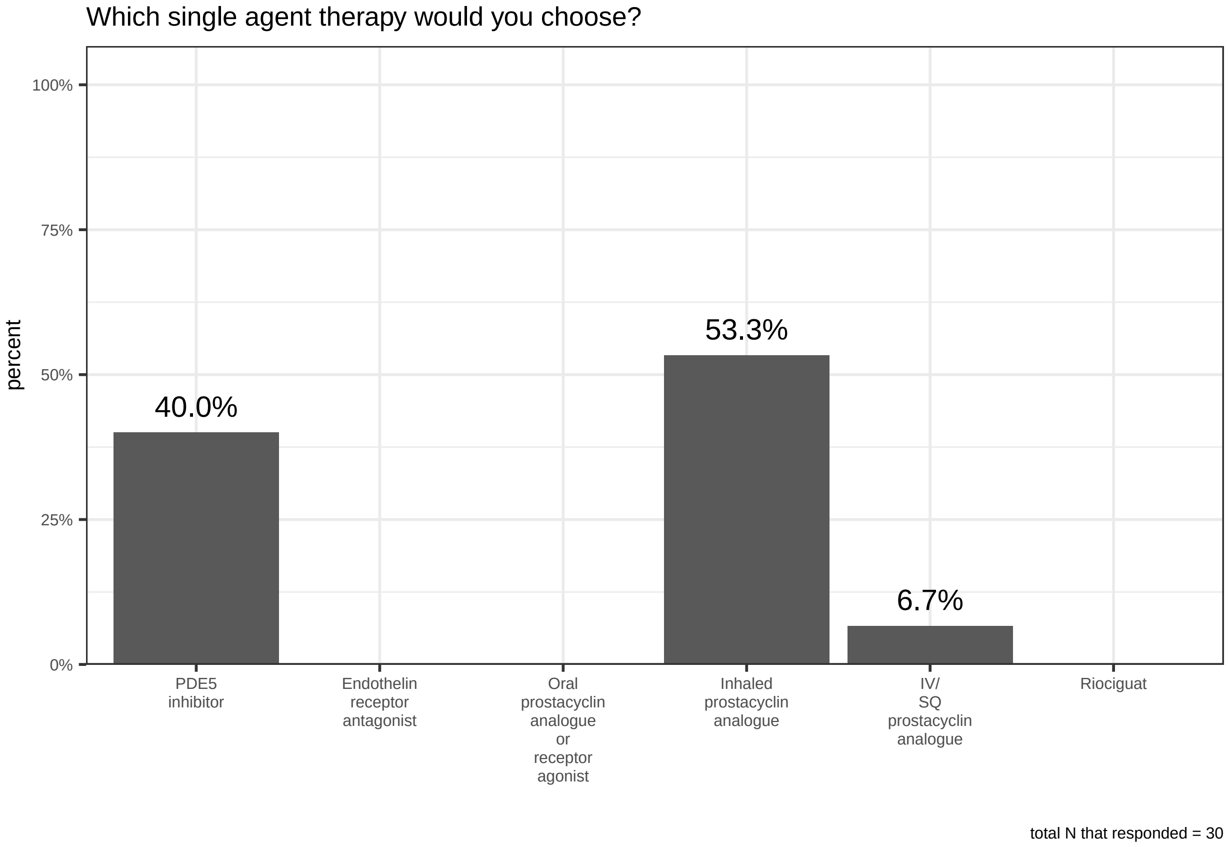
Which single agent therapy would you choose?
percent
100%
75%
50%
25%
0%
40.0%
53.3%
6.7%
PDE5 inhibitor
Endothelin receptor antagonist
Oral prostacyclin analogue or receptor agonist
Inhaled prostacyclin analogue
IV/ SQ prostacyclin analogue
Riociguat
total N that responded = 30

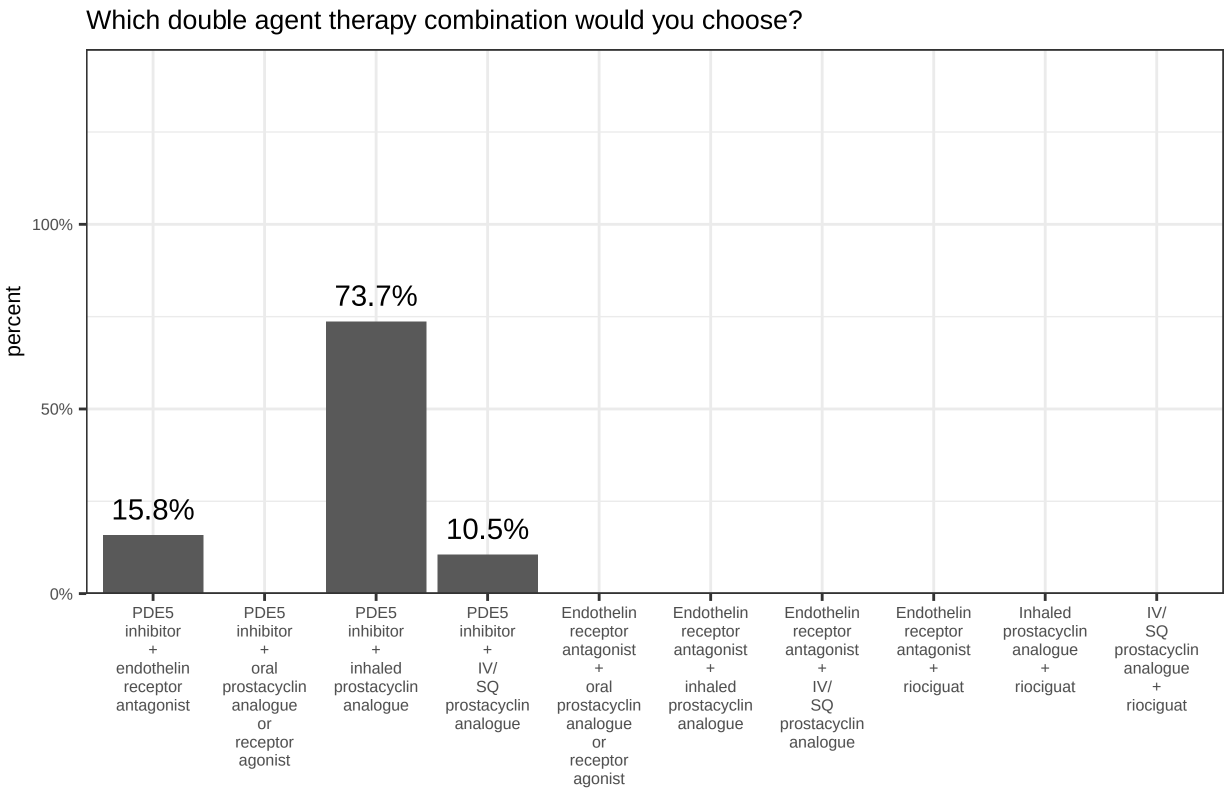

total N that responded = 19

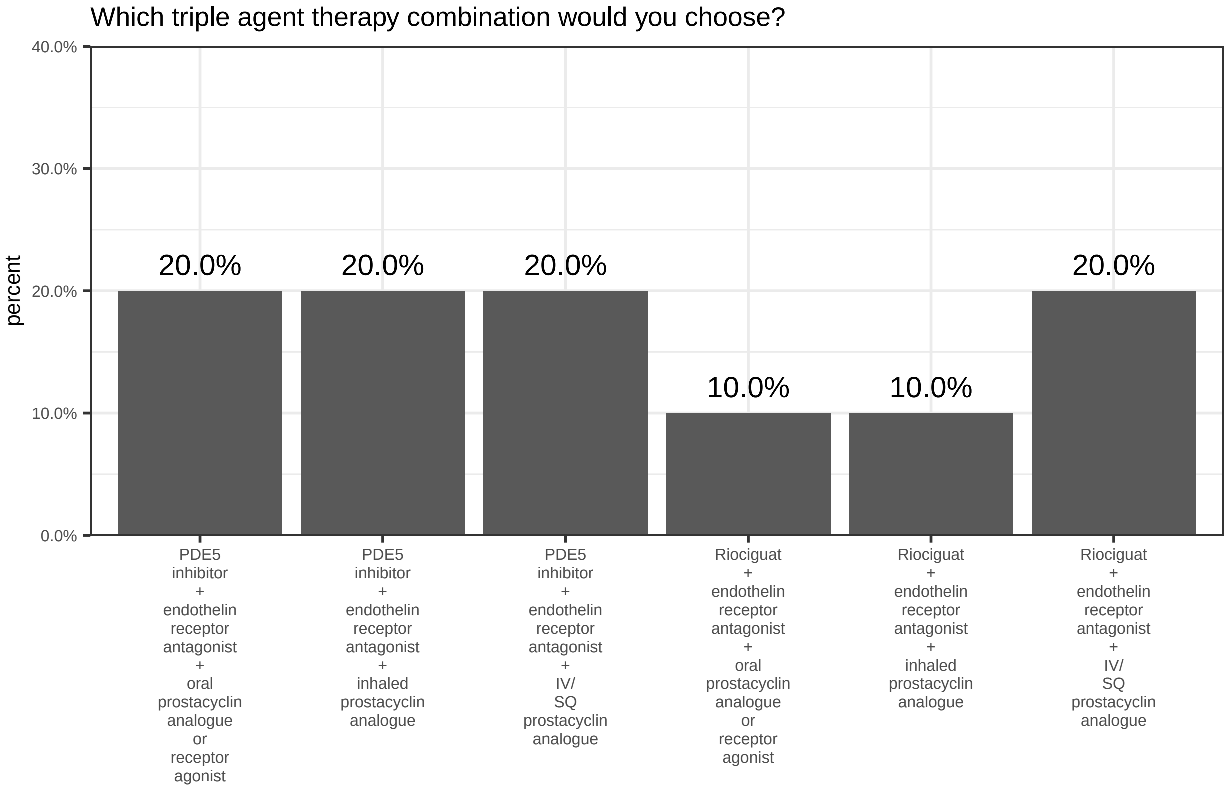

total N that responded = 10

Q13 − case 5

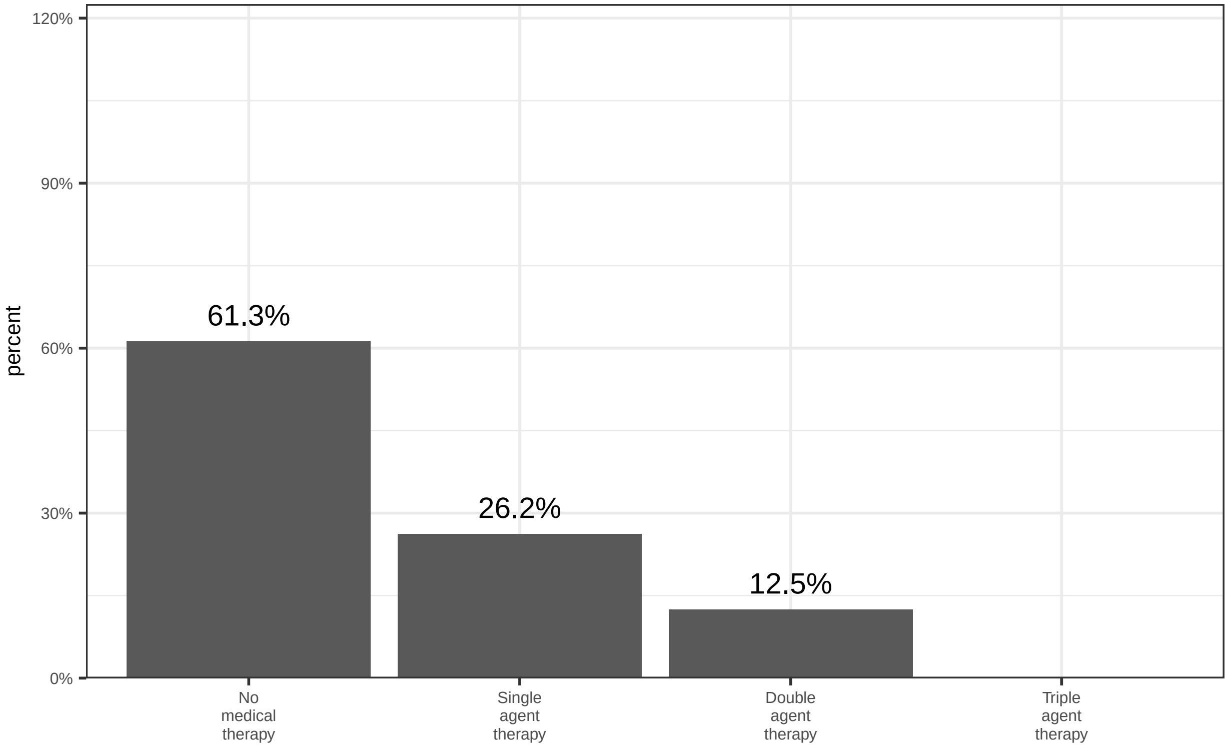

total N that responded = 80

Which single agent therapy would you choose?

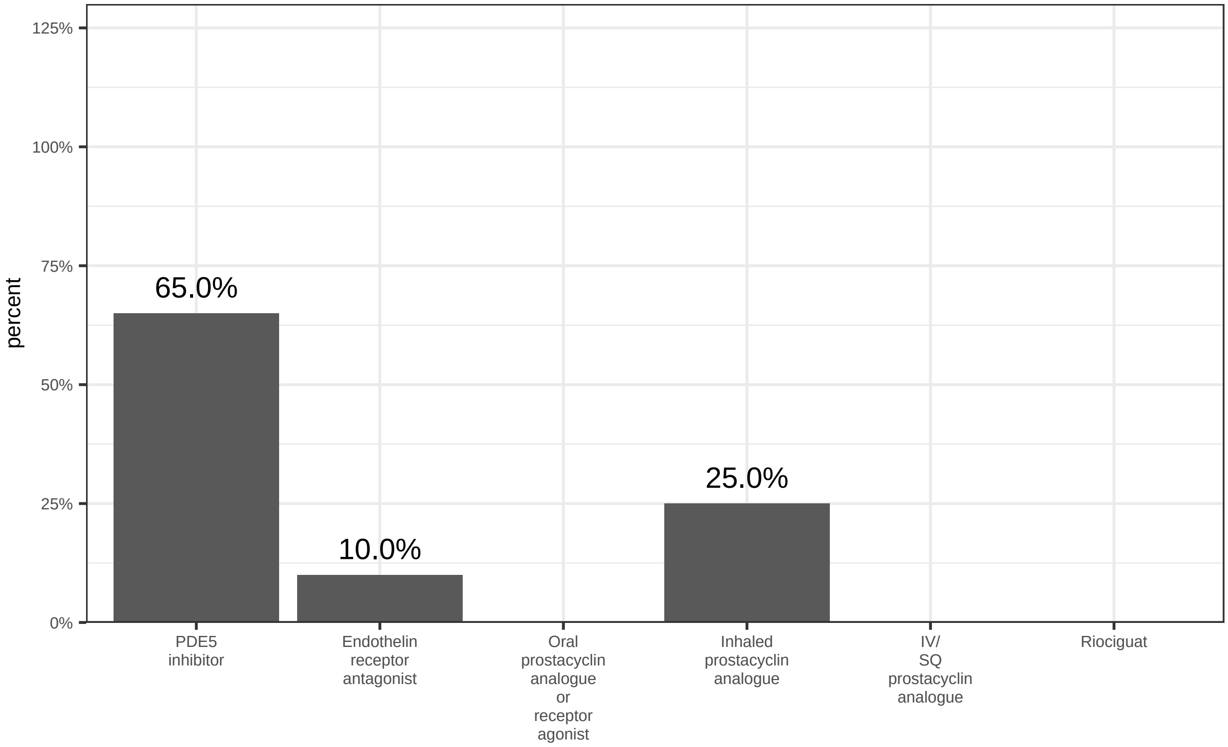

total N that responded = 20

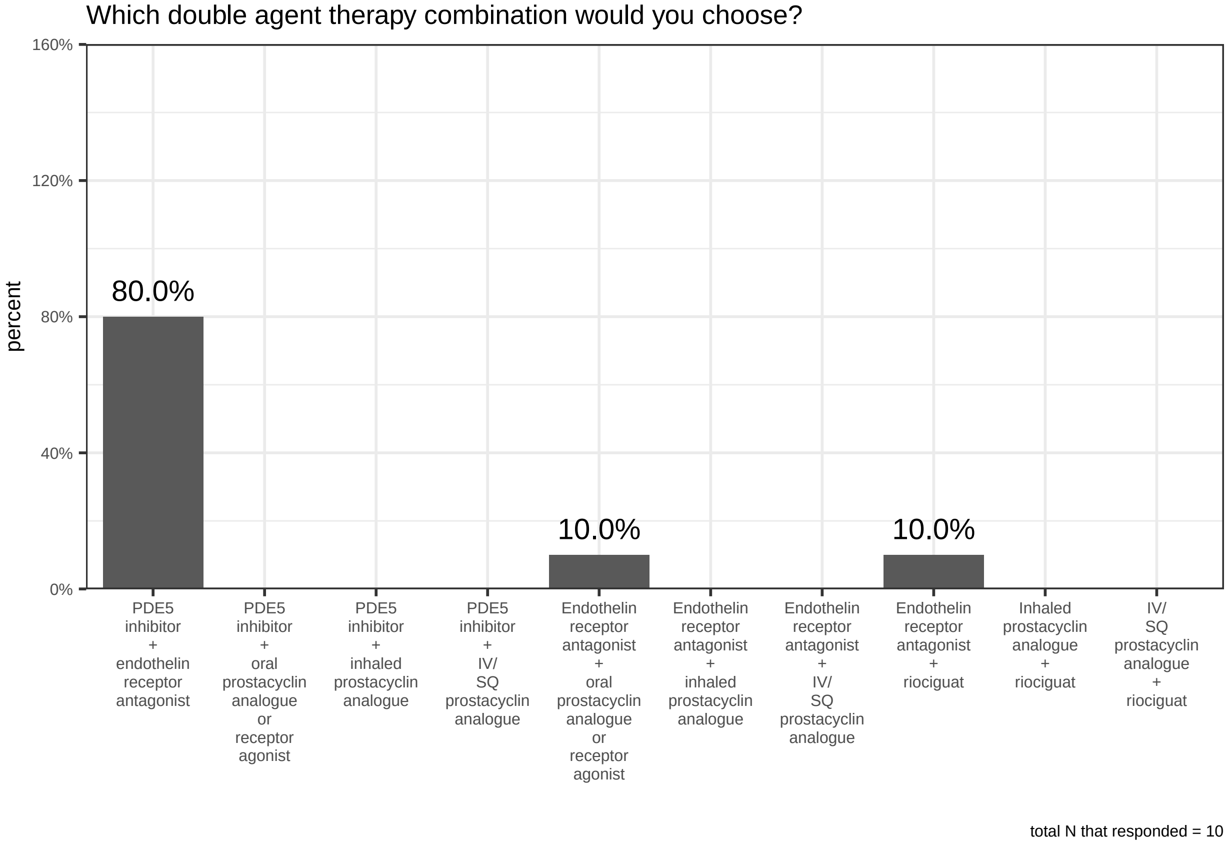

Which double agent therapy combination would you choose?
percent
160%
120%
80%
40%
0%
80.0%
10.0%
10.0%
PDE5 inhibitor + endothelin receptor antagonist
PDE5 inhibitor + oral prostacyclin analogue or receptor agonist
PDE5 inhibitor + inhaled prostacyclin analogue
PDE5 inhibitor + IV/ SQ prostacyclin analogue
Endothelin receptor antagonist + oral prostacyclin analogue or receptor agonist
Endothelin receptor antagonist + inhaled prostacyclin analogue
Endothelin receptor antagonist + IV/ SQ prostacyclin analogue
Endothelin receptor antagonist + riociguat
Inhaled prostacyclin analogue + riociguat
IV/ SQ prostacyclin analogue + riociguat
total N that responded = 10

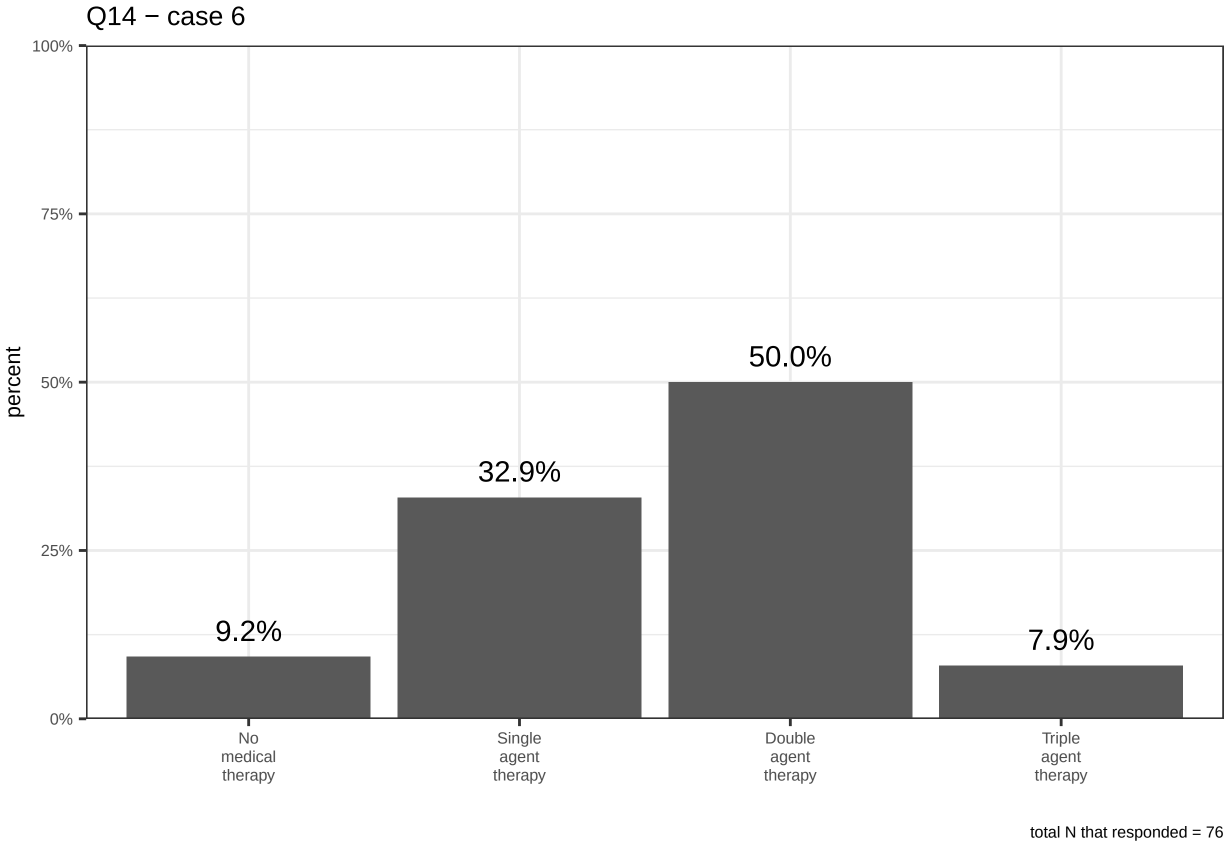

Q14 − case 6
percent
100%
75%
50%
25%
0%
9.2%
32.9%
50.0%
7.9%
No medical therapy
Single agent therapy
Double agent therapy
Triple agent therapy
total N that responded = 76

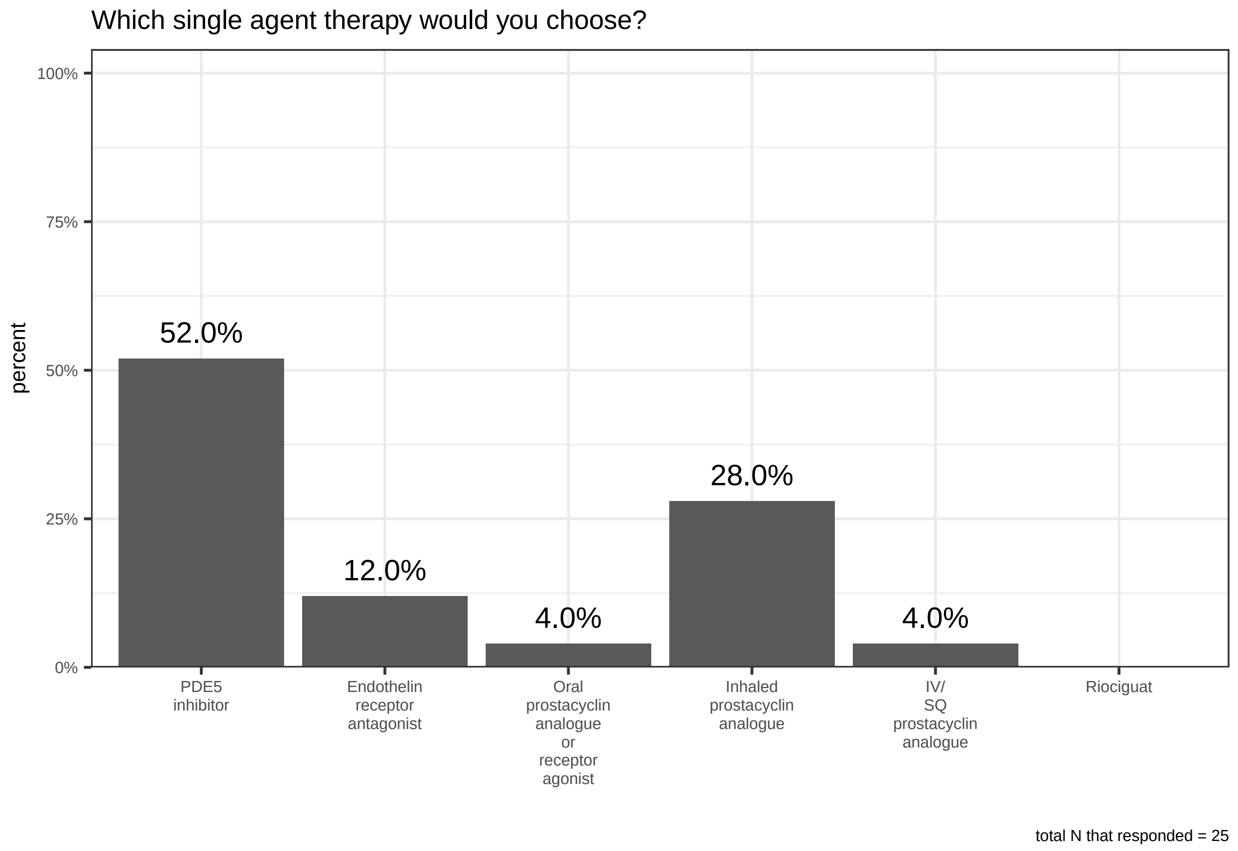
Which single agent therapy would you choose?
percent
100%
75%
50%
25%
0%
52.0%
12.0%
4.0%
28.0%
4.0%
PDE5 inhibitor
Endothelin receptor antagonist
Oral prostacyclin analogue or receptor agonist
Inhaled prostacyclin analogue
IV/ SQ prostacyclin analogue
Riociguat
total N that responded = 25

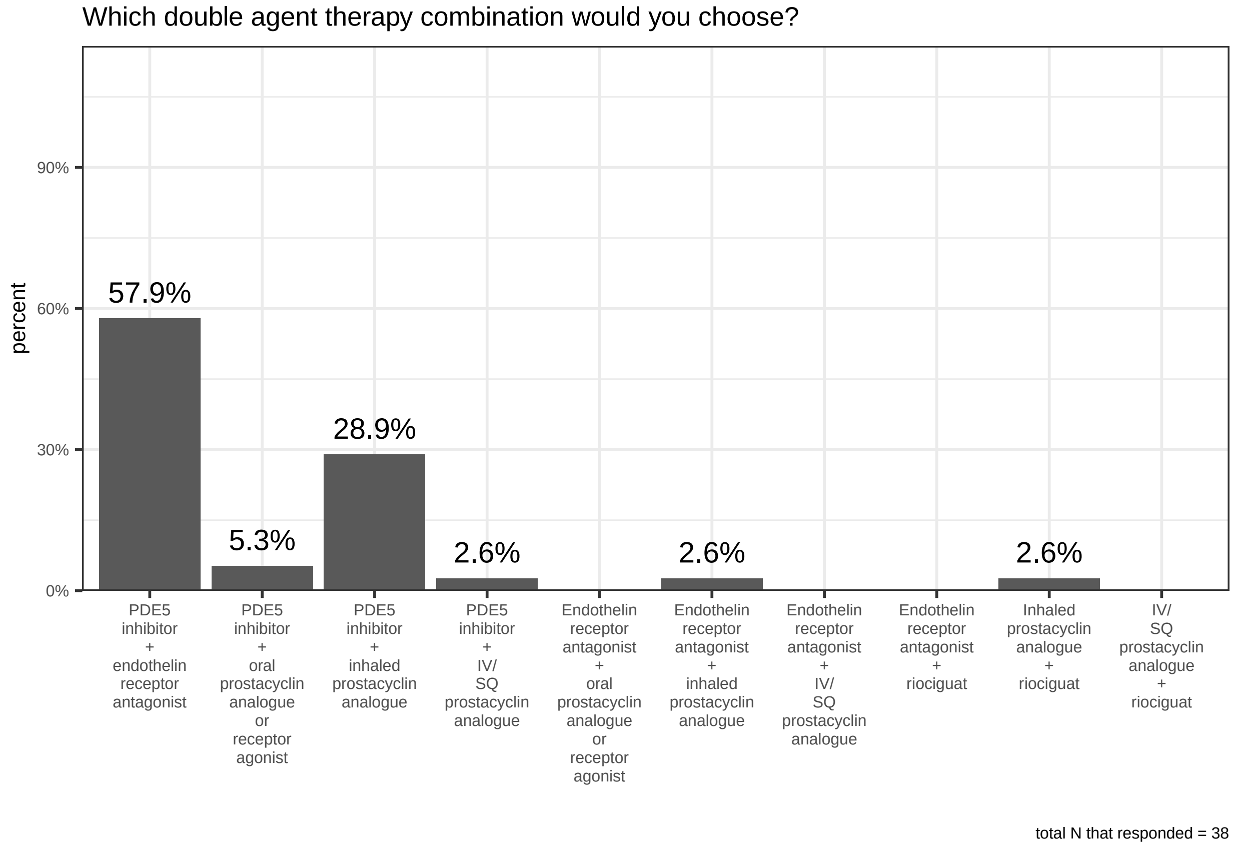
Which double agent therapy combination would you choose?
percent
90%
60%
30%
0%
57.9%
5.3%
28.9%
2.6%
2.6%
2.6%
PDE5 inhibitor + endothelin receptor antagonist
PDE5 inhibitor + oral prostacyclin analogue or receptor agonist
PDE5 inhibitor + inhaled prostacyclin analogue
PDE5 inhibitor + IV/ SQ prostacyclin analogue
Endothelin receptor antagonist + oral prostacyclin analogue or receptor agonist
Endothelin receptor antagonist + inhaled prostacyclin analogue
Endothelin receptor antagonist + IV/ SQ prostacyclin analogue
Endothelin receptor antagonist + riociguat
Inhaled prostacyclin analogue + riociguat
IV/ SQ prostacyclin analogue + riociguat
total N that responded = 38

Which triple agent therapy combination would you choose?

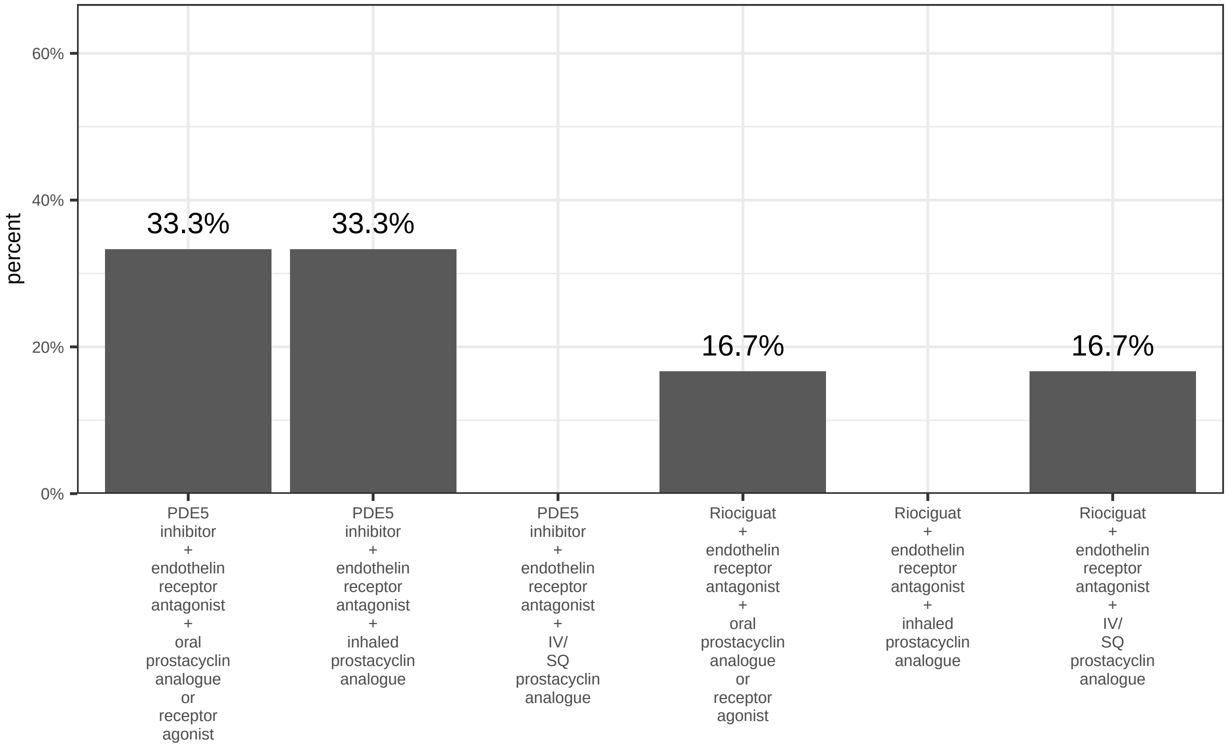

total N that responded = 6

Q15 − case 7

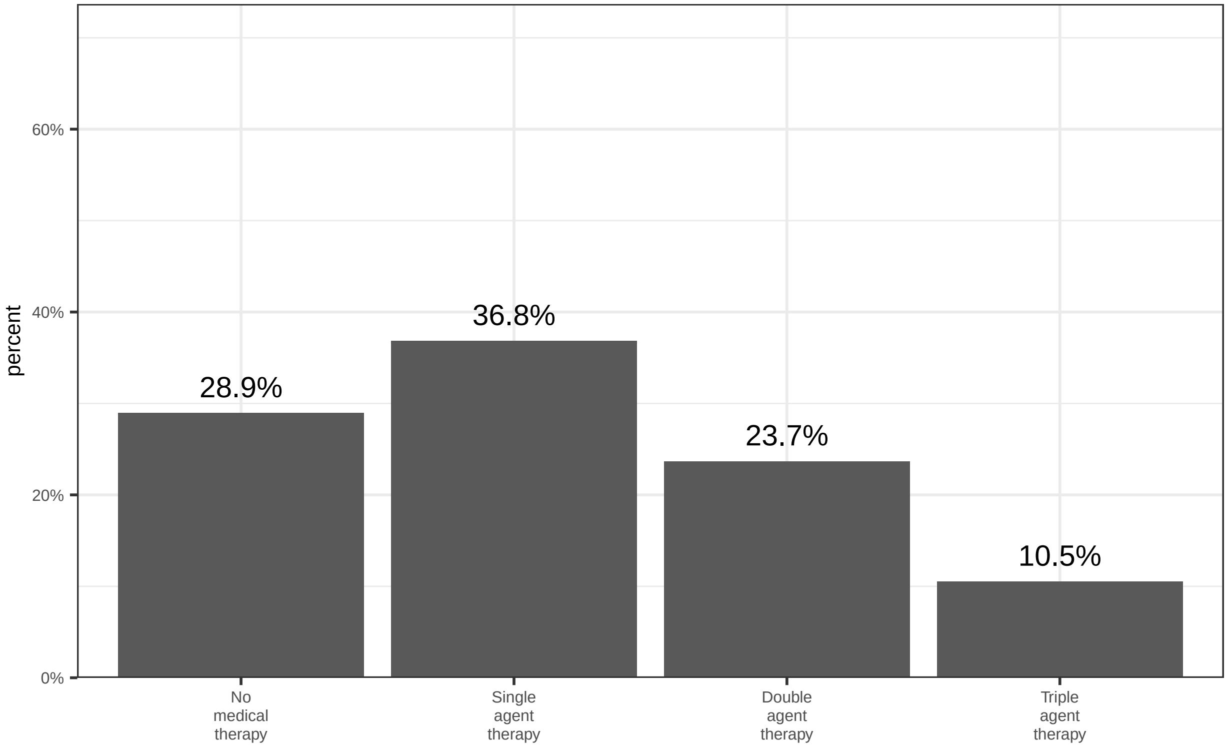

total N that responded = 76

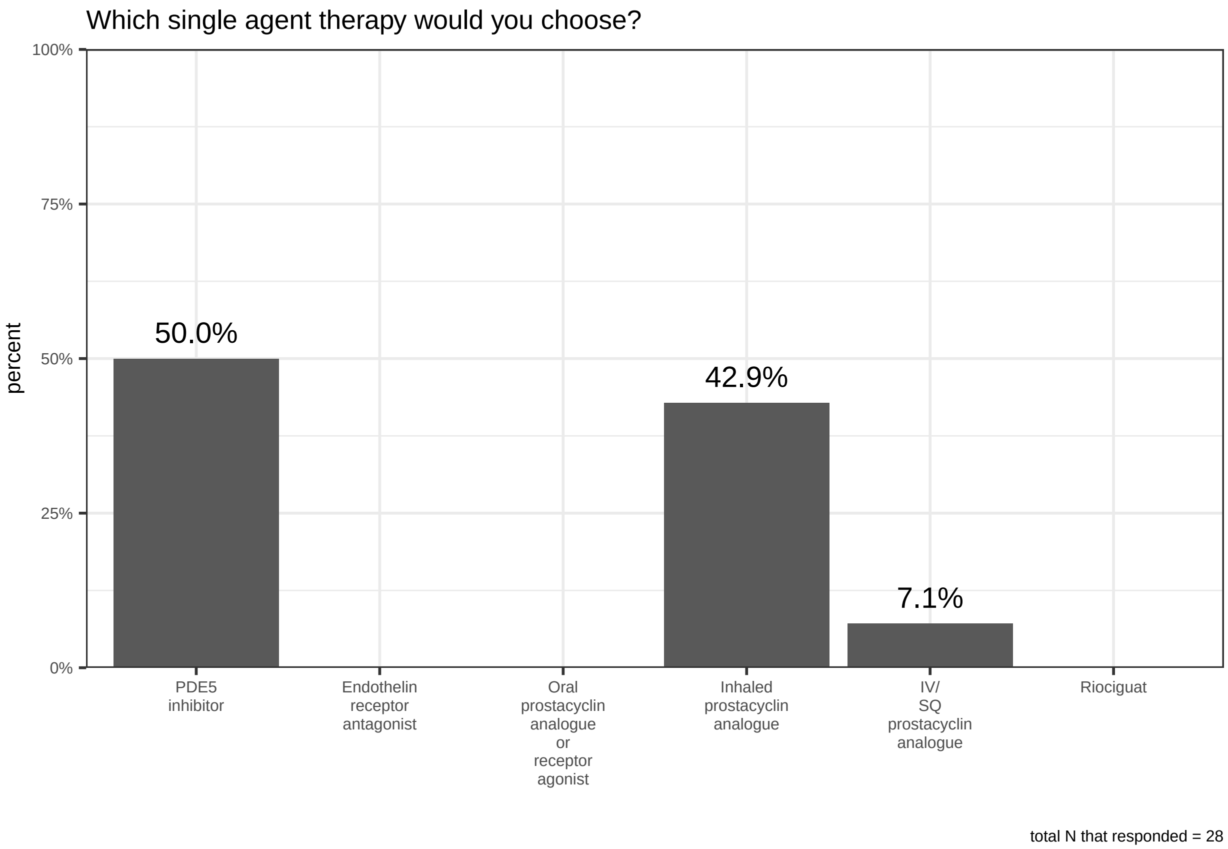
Which single agent therapy would you choose?
100%
75%
50%
25%
0%
percent
50.0%
42.9%
7.1%
PDE5 inhibitor
Endothelin receptor antagonist
Oral prostacyclin analogue or receptor agonist
Inhaled prostacyclin analogue
IV/ SQ prostacyclin analogue
Riociguat
total N that responded = 28

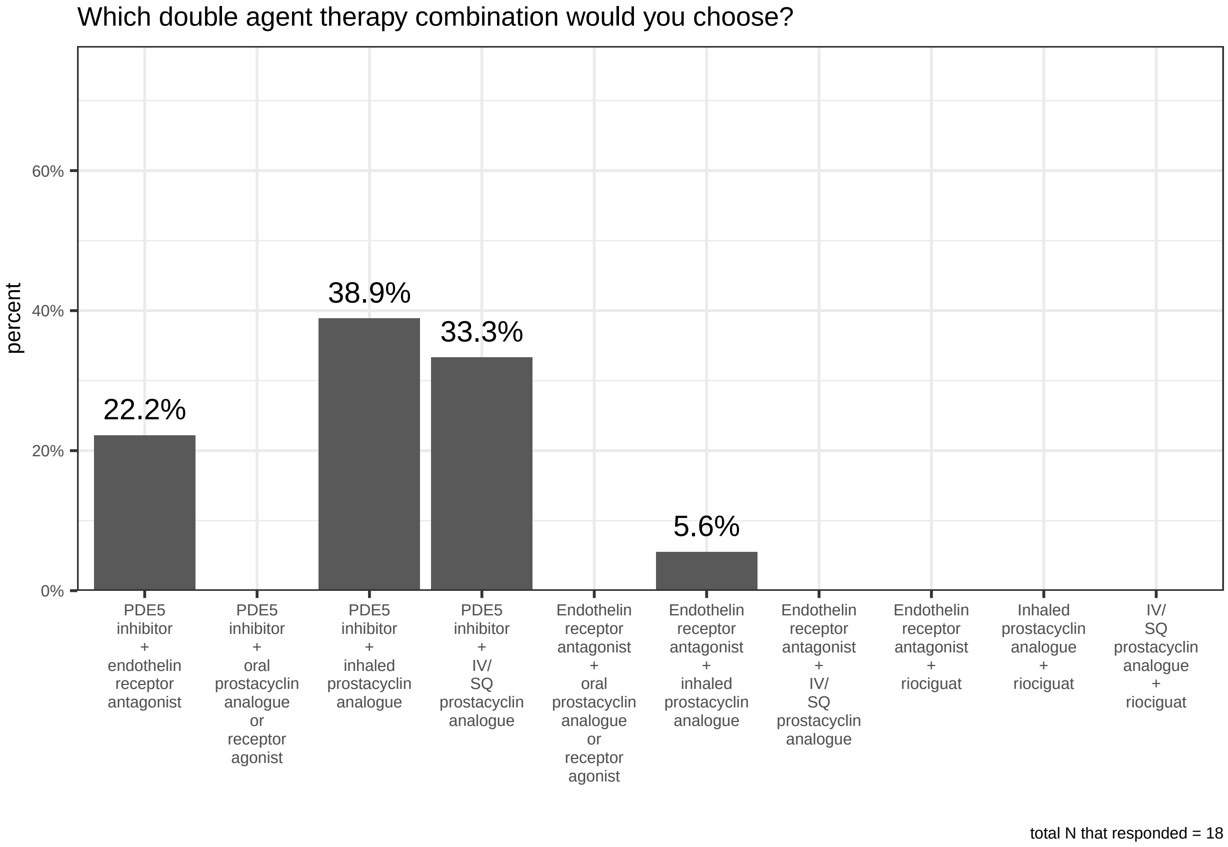
Which double agent therapy combination would you choose?
60%
40%
20%
0%
percent
22.2%
38.9%
33.3%
5.6%
PDE5 inhibitor + endothelin receptor antagonist
PDE5 inhibitor + oral prostacyclin analogue or receptor agonist
PDE5 inhibitor + inhaled prostacyclin analogue
PDE5 inhibitor + IV/ SQ prostacyclin analogue
Endothelin receptor antagonist + oral prostacyclin analogue or receptor agonist
Endothelin receptor antagonist + inhaled prostacyclin analogue
Endothelin receptor antagonist + IV/ SQ prostacyclin analogue
Endothelin receptor antagonist + riociguat
Inhaled prostacyclin analogue + riociguat
IV/ SQ prostacyclin analogue + riociguat
total N that responded = 18

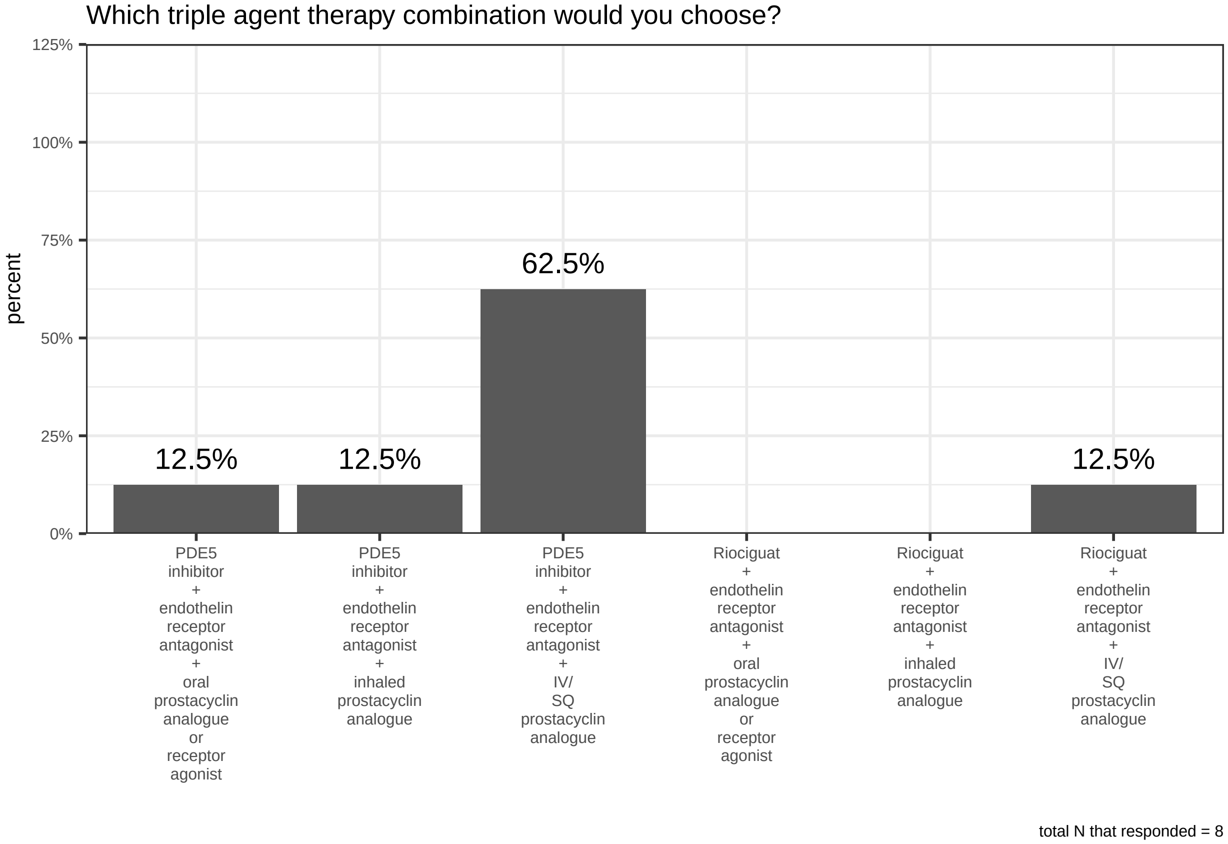

table of Fleiss' Kappa for 76 raters (all cases, IPF cases only, COPD cases only)

```
##      num_raters kap_all kap_ipf kap_copd
## [1,]         76    0.11    0.13      0.1
```

table of Fleiss' Kappa for 70 US only raters (all cases, IPF cases only, COPD cases only)

```
##      num_raters kap_us_all kap_us_ipf kap_us_copd
## [1,]         70       0.11       0.12         0.1
```

table of Fleiss' Kappa for 53 Pulmonologist raters (all cases, IPF cases only, COPD cases only)

```
##      num_raters kap_all_pulm kap_ipf_pulm kap_copd_pulm
## [1,]         53         0.13         0.14          0.12
```

table of Fleiss' Kappa for 22 Cardiologist raters (all cases, IPF cases only, COPD cases only)

```
##      num_raters kap_all_cardio kap_ipf_cardio kap_copd_cardio
## [1,]         22           0.07           0.08            0.05
```

below are for binary treatment or no treatment

table of Fleiss' Kappa for 76 raters (all cases, IPF cases only, COPD cases only)

```
##      num_raters kap_bin_all kap_bin_ipf kap_bin_copd
## [1,]         76        0.23        0.27         0.21
```

table of Fleiss' Kappa for 70 US only raters (all cases, IPF cases only, COPD cases only)

```
##      num_raters kap_bin_us_all kap_bin_us_ipf kap_bin_us_copd
## [1,]         70           0.24           0.28            0.23
```

table of Fleiss' kap_binpa for 53 Pulmonologist raters (all cases, IPF cases only, COPD cases only)

```
##      num_raters kap_bin_all_pulm kap_bin_ipf_pulm kap_bin_copd_pulm
## [1,]         53             0.25             0.31              0.23
```

table of Fleiss' kap_binpa for 22 Cardiologist raters (all cases, IPF cases only, COPD cases only)

```
##      num_raters kap_bin_all_cardio kap_bin_ipf_cardio kap_bin_copd_cardio
## [1,]         22               0.14               0.14                0.12
```

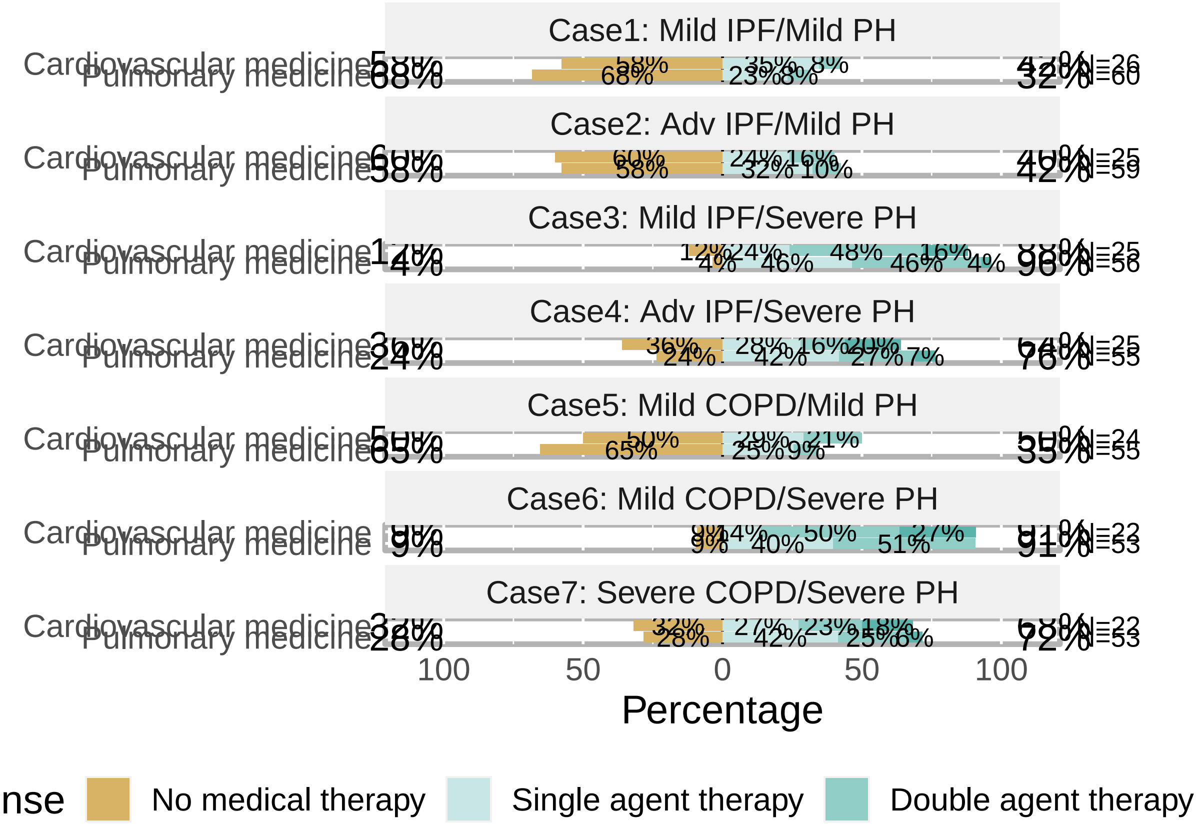

Supplement: Supplementary file 2 [file Data_Sheet_2.PDF]
